# Beyond aromatherapy: can essential oil loaded nanocarriers revolutionize cancer treatment?

**DOI:** 10.1039/d4na00678j

**Published:** 2024-09-27

**Authors:** Obaydah Abd Alkader Alabrahim, Jude Majed Lababidi, Wolfgang Fritzsche, Hassan Mohamed El-Said Azzazy

**Affiliations:** a Department of Chemistry, School of Sciences & Engineering, The American University in Cairo AUC Avenue, SSE # 1184, P.O. Box 74 New Cairo 11835 Egypt Obaydah.alabrahim@aucegypt.edu jlababidi@aucegypt.edu hazzazy@aucegypt.edu; b Department of Nanobiophotonics, Leibniz Institute of Photonic Technology Albert Einstein Str. 9 Jena 07745 Germany

## Abstract

Cancer, a complex global health burden, necessitates the development of innovative therapeutic strategies. While chemotherapy remains the primary treatment approach, its severe side effects and chemoresistance drive the search for novel alternatives. Essential oils (EOs), consisting of diverse bioactive phytochemicals, offer promise as anticancer agents. However, their limitations, such as instability, limited bioavailability, and non-specific targeting, hinder their therapeutic potential. These challenges were circumvented by utilizing nanoparticles and nanosystems as efficient delivery platforms for EOs. This review highlights the accumulating evidence based on loading EOs into several nanocarriers, including polymeric nanoparticles, nanoemulsions, nanofibers, lipid-based nanocapsules and nanostructures, niosomes, and liposomes, as effective anticancer regimens. It covers extraction and chemical composition of EOs, their mechanisms of action, and targeting strategies to various tumors. Additionally, it delves into the diverse landscape of nanocarriers, including their advantages and considerations for cancer targeting and EO encapsulation. The effectiveness of EO-loaded nanocarriers in cancer targeting and treatment is examined, highlighting enhanced cellular uptake, controlled drug release, and improved therapeutic efficacy. Finally, the review addresses existing challenges and future perspectives, emphasizing the potential for clinical translation and personalized medicine approaches.

## Abstract 2

Loading essential oils in novel nanocarriers enhances their activities against cancer, a complex global health burden.

## Graphical abstract

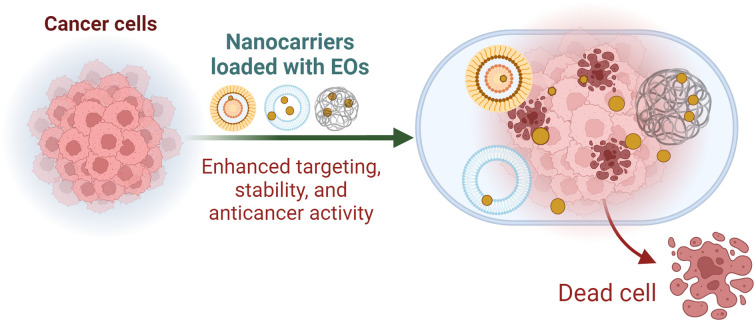

## 1. Introduction

Cancer is still considered the leading cause of death worldwide with a fast growth in the mortality and incidence rates in 112 countries (the Global Cancer Observatory (GLOBOCAN), 2020).^1^ Female breast cancer was responsible for the highest number of diagnosed cases (11.7%), followed by lung (11.4%), colorectal (10%), prostate (7.3%), and stomach (5.6%) cancers.^1^ Different criteria were used to define the most effective treatment approaches for different cancers whilst relying mainly on the tumor type/subtype, grade, and stage. Nevertheless, the general treatment approach for treating almost all cancers includes surgery, radiotherapy, and chemotherapy.^2–6^ This approach is still hindered by high relapse rates and chemoresistance, in addition to severe and chronic toxicities affecting several vital organs.^7–10^

Many natural extracts have been favorably exploited for targeting various cancers, including essential oils (EOs) attributed to their outstanding medicinal and therapeutic properties (*e.g.*, anticancer, antimicrobial, anti-inflammatory, spasmolytic, sedative, *etc*.).^11,12^ Different malignancies have shown great recession following their treatment with EOs of various plants.^13–17^ Several mechanisms have been recognized for the preventive and therapeutic anticancer activities of EOs, including antimutagenic, antiproliferative, and antioxidant mechanisms.^13–17^ However, a few challenges still hinder EO exploitation for cancer targeting, mainly for clinical applications. These challenges include poor bioavailability, solubility, chemical instability, high volatility, and humidity and light sensitivity of EOs.^18^

Nanotechnology has provided promising solutions by utilizing various nanocarriers as effective drug delivery systems for EO delivery against various tumor tissues. Drug delivery systems fabricated on nanoformulations have depicted superior characteristics.^19–26^

These characteristics include sustained release, greater EO permeability against tumors, improved EO bioavailability and solubility, and eventually improved therapeutic efficacy.^27^

In light of this context, this review provides an introduction to EO extraction, chemical composition, nanotechnology, common classification and properties of nanomaterials, and nanoplatforms as promising drug delivery carriers. More importantly, the core focus of the current review involved state-of-the-art novel nanosystems reported in the most recent studies as promising nanocarriers for augmenting the anticancer activities of EOs against different tumor cells and tissues.

## 2. Chemotherapy

Chemotherapeutic drugs inhibit the proliferation and growth of cancer cells, either by a direct or an indirect pathway, and are usually classified in relation to their mechanism of action, including mitotic inhibitors, topoisomerase inhibitors, protein kinase inhibitors, antibiotics, antimetabolites, and alkylating chemotherapeutic drugs.^28^ For instance, many groups of chemotherapeutic drugs could be used for targeting breast malignancies, comprising taxanes (*e.g*., paclitaxel), alkylating agents (*e.g*., cyclophosphamide), and antimetabolites (*e.g*., 5-fluorouracil (5FU)).^7,26,29,30^

**Fig. 1:**
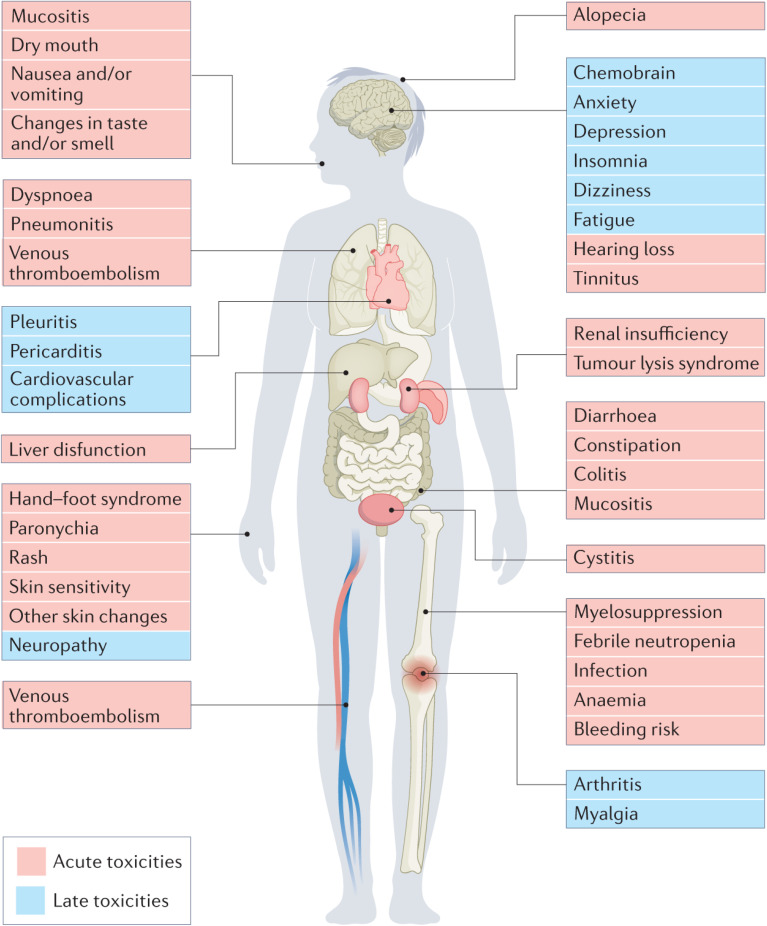
Common severe and chronic toxicities associated with chemotherapy, affecting all organ systems.^10^ Reproduced with permission from ref. 10. Copyright 2022 Springer Nature.

Nevertheless, severe and chronic toxicities in addition to chemoresistance characterize the main downsides of chemotherapy for treating all cancers, negatively impacting all organ systems.^7–10^ Early side effects of treatment may include hair loss, blood cell deficiencies, peripheral neuropathy, and musculoskeletal disorders. Alongside the high rates of tumor recurrence and relapse, long-term toxicities can include thromboembolism, infertility, cardiomyopathy, psychological abnormalities, gastrointestinal disorders, neurotoxicity, and bone and joint degeneration^7–10^ (Fig. 1).

Several mechanisms have been established behind chemoresistance development causing multiple resistance against chemotherapeutics, where tumor cells can escape and develop resistance against these drugs. These mechanisms include altering the expression of targeted cells, increasing the DNA repair rate, inactivating chemotherapeutics, increasing *P*-glycoprotein expression. and changing certain apoptotic pathways of tumor cells.^31–36^

## 3. Essential oils

Essential oils (EOs) are a diverse group of secondary metabolites consisting of complex mixtures. These complexes are characterized by their low molecular weight, strong scent, and volatility, and are primarily composed of volatile terpenes and hydrocarbons.^37^ The diversity of EOs in their parent plants can be extremely influenced by the plant part utilized as a raw material. However, EOs are usually responsible for only a small portion of the entire dried plant (∼5%) and might be extracted from several plant parts such as seeds, fruits, gums, and leaves.^38–41^

Due to their diverse medicinal and antiseptic properties, EOs hold immense potential for pharmaceutical and biomedical applications. These properties include a range of benefits like anti-inflammatory, pain-relieving, and even anticancer effects. Additionally, EOs demonstrate antifungal, antibacterial, and antiviral activities, making them valuable antiseptics.^12^

### 3.1. EO extraction methods

EOs are extracted from their respective plant sources *via* different extraction methods. Conventional methods include solvent extraction and distillation. Recent extraction approaches normally refer to two common methods, microwave-assisted extraction and supercritical fluid extraction. These recent methods offer a significant advantage over traditional methods as they minimize oxidation and breakdown of the extracted compounds. This is particularly beneficial for preserving the delicate fragrances extracted from flowers.^42,43^

**Fig. 2:**
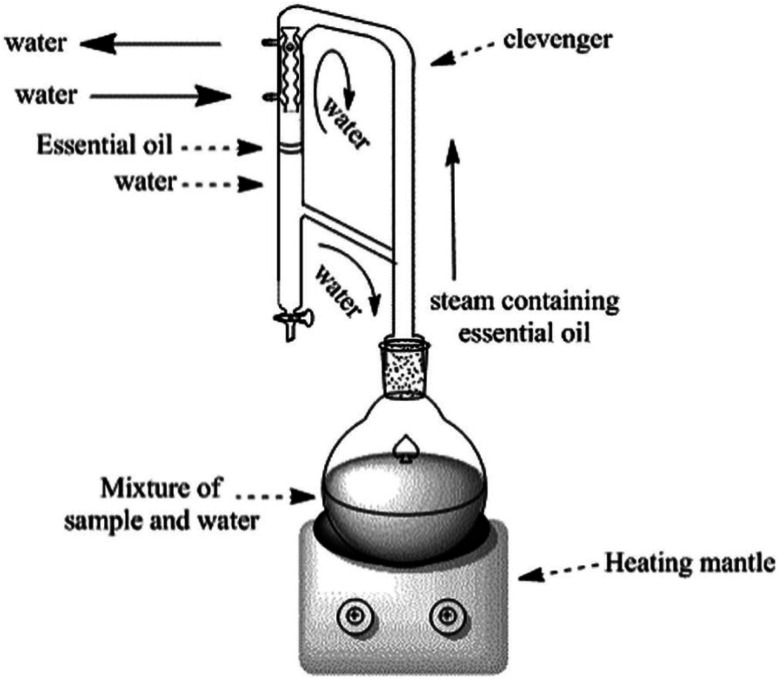
Clevenger apparatus system for EO extraction through hydrodistillation. The figure elucidates the basic components of Clevenger apparatus in addition to the main steps accomplished during the hydrodistillation extraction.^44^ Reproduced with permission from ref. 44 Copyright 2017 Elsevier.

The volatile and aromatic nature of EOs makes hydrodistillation, using the Clevenger apparatus (Fig. 2), a particularly suitable method for small-scale (laboratory-scale) extraction of EOs. Nevertheless, steam distillation and solvent extraction methods have been well-acknowledged and conventionally utilized for larger scale processes (*i.e*., industrial scale). However, the higher toxicity accompanying the use of organic solvents has restricted the use of the solvent extraction method for critical and sensitive applications in food, medical, and pharmaceutical industries. Hence, hydrodistillation, supercritical fluid extraction, and microwave extraction have been preferably utilized for EO extraction due to their established properties of safety, efficiency, and sustainability.^42,43,45^

#### 3.1.1. Solvent extraction

EOs with thermosensitive components are usually extracted using solvent extraction. Throughout the extraction process, the plant can be placed in a tank mixed with suitable organic solvent(s) that can dissolve the material. Subsequently, the obtained liquid mixture, containing EOs alongside other natural components extracted, can be withdrawn to be further subjected to a filtration step followed by a distillation process. Common solvents utilized include methanol, ethanol, petroleum ether, and hexane. Despite its widespread use, this method has several drawbacks such as toxic residues, volatile component loss, and prolonged extraction time.^46^

#### 3.1.2. Steam distillation

**Fig. 3:**
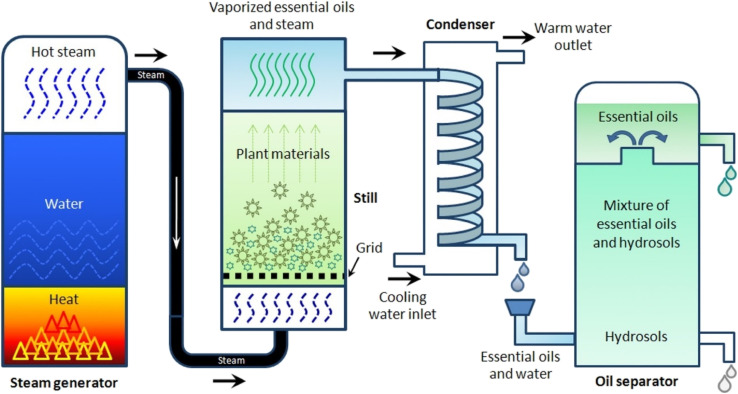
Schematic representation depicts the steam distillation extraction method (process steps and main parts) utilized for EO extraction.^47^ Reproduced with permission from ref. 47 Copyright 2014 John Wiley & Sons.

The majority of EOs (93%) are extracted using this method. Throughout the distillation extraction process, the source material is subjected to steam, releasing the EOs. Consequently, the steam that carries the EOs is condensed *via* a cooling system, and the obtained condensate can be gathered inside a separation tank (Fig. 3).^43,46^

#### 3.1.3. Supercritical fluid extraction

**Fig. 4:**
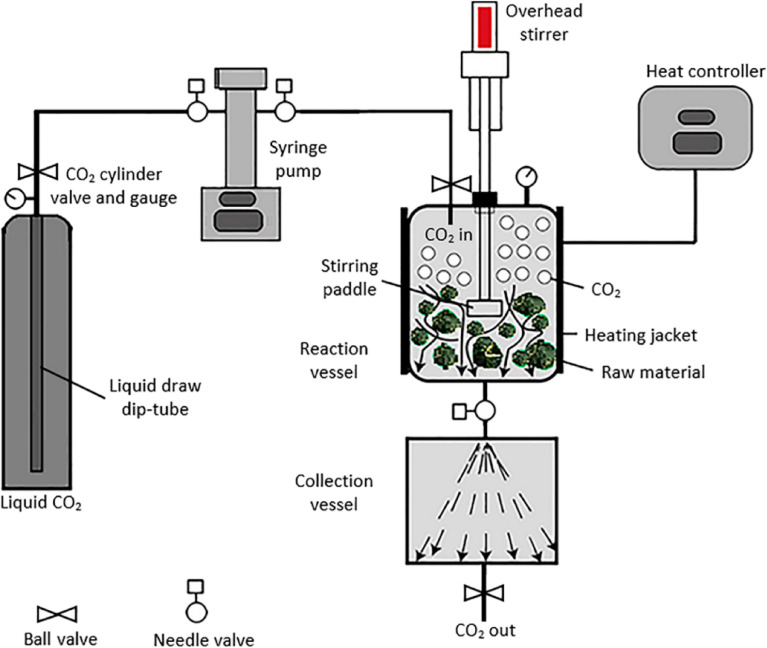
The standard system of a supercritical fluid device.^48^ Reproduced from ref. 48 Copyright 2017 MDPI.

Supercritical extraction exploits a supercritical liquid of CO_2_ for EO extraction (Fig. 4).^48^ Throughout the extraction process, the fluid is subjected to repeating cycles of compression and decompression for which the fluid can be heated and compressed until reaching the supercritical state of CO_2_. Consequently, the supercritical fluid is allowed to go through the provided plant material to generate volatile substances and other components. Subsequently, the obtained mixture can be supplied into two separators, where a decompression step could be applied to separate the CO_2_ from the plant extract(s).^43^

The following table (Table 1) reveals the extraction of some EOs from their corresponding plant materials using the four common extraction methods: steam distillation, solvent extraction, supercritical solvent extraction, and hydrodistillation.

**Table 1:** Examples of EOs extracted by common extraction methods

Extraction method	Plant materials
Steam distillation	Clove (*Eugenia caryophyllata*);^49^ Eucalyptus (*Eucalyptus citriodora* and *Camaldulensis*);^50,51^ Peppermint (*Mentha peperita* L.);^50^ Rose geranium (*Pelargonium* species);^52^ Patchouli (*Pogostemon cablin*);^53^ Thyme (*Thymus kotschyanus*);^54^ Rosemary (*Rosmarinus officinalis*).^55–57^ Cumin, cloves, and peppermint;^56^ Lavender;^58,59^ Mint (*Mentha peperita* L. and *Mentha spicada* L);^60^ Basil (*Ocimum basilicum* L);^57^*Baccharises* (*Baccharis dentata*, *B. anomala*, and *B. uncinella*);^61^ Lavender (*Lavandula dentata* L);^57^ Peppermint;^62^ Orange (*Citrus sinensis*);^63^ Germander (*Teucrium orientale*);^64^ Anise (*Pimpinella anisum*);^55^ Fennel (*Foeniculum vulgare*)^55^
Hydrodistillation	Sage (*Salvia officinalis*);^65^ Cumin (*Cuminum cyminum*);^66^ Pomelo (*Citrus maxima*);^67^ Caraway (*Carum carvi*);^68^ Lavender (*Lavandula angustifolia*);^65^ Bhaktiary savory (*Satureja bachtiarica Bunge*);^69^ Cypress (*Cupressus sempervirens* L);^70^ Anise hyssop (*Lophantus anisatus*);^65^ Clove (*Eugenia caryophyllata*);^49^ Rosemary (*Rosmarinus officinalis* L);^71–73^ Hyssop (*Hyssopus officinalis*);^65^ Sweet bay (*Laurus nobilis* L);^74^*O. vulgare* L. subspecies *hirtum*;^75^ Thyme (*Thymus vulgaris*);^76^ Marjoram (*Majorana hortensis*);^65^ Rose geranium (*Pelargonium* species);^52^ Germander (*Teucrium orientale*);^64^ Oregano (*Origanum vulgare* L);^77^ Catnip (*Nepeta cataria*)^65^
Solvent extraction	*Thymus longicaulis* subspecies *longicaulis* variety *longicaulis*;^78^ Apiaceae (*Ptychotis verticillata*);^79^ Chasteberry (*Vitexagnuscastus*);^80^ Lemon (*Citrus limon*).^81^ Sage (*Salvia officinalis*).^82,83^
Supercritical solvent extraction	Marjoram (*Majorana hortensis*);^65^ Rosemary (*Rosmarinus officinalis*);^55,73^ Coriander (*Coriandrum sativum*);^84^ Fennel (*Foeniculum vulgare*);^55,85^ Lemon (*Citrus limon*);^86^ Catnip (*Nepeta cataria*);^65^ Thyme (*Thymus vulgaris*);^65^ Hyssop (*Hyssopus officinalis* L);^65^ Patchouli (*Pogostemon cablin*);^53^ Cumin (*Cuminum cyminum*);^66,85^ Chamomile (*Chamomilla recutita* L. Rauschert and *Matricaria chamomilla*);^87,88^ Lavender (*Lavandula Stoechas* subspecies *Cariensis Boiss* and *Lavandula angustifolia*);^65,88,89^ Oregano (*Origanum virens* and *O. vulgare*);^65,88,90^ Carrot (*Daucus carrota*);^91^ Pennyroyal;^88,92^ Sage (*Suluiu oficinalis* and *Salvia officinalis*).^65,88,93,94^ Anise (*Pimpinella anisum*);^55,85^ Anise hyssop (*Lophantus anisatus*);^65^ Clove (*Eugenia caryophyllata*)^49^

### 3.2. Key phytochemicals of EOs

**Fig. 5:**
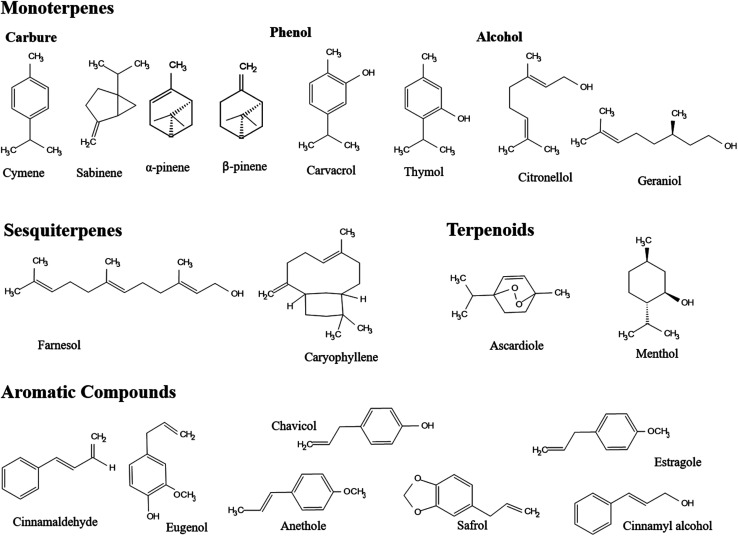
Chemical structures of several compounds, assigned to different groups, present in EOs, including terpenes (*e.g.*, monoterpenes and sesquiterpenes), terpenoids, and aromatic compounds.

The chemical composition of EOs encompasses a group of diverse and yet complex natural compounds present in huge numbers among various plant species.^95^ However, two distinct groups of components featured in EOs have contributed to their unique therapeutic properties.^96^ Terpenes represent the prime group of compounds which are made of a combination of isoprene (5 carbon-based units) and terpenoids (contain oxygen atom(s) in their structures).^37^ Terpenoids, in particular, incorporate various functional compounds in EOs such as phenols, alcohols, esters, ketones, ethers, acids, and aldehydes.^40^ The hydrocarbon terpene group signifies more than 80% of the plant EOs such as monoterpenes (10 carbon-based units) and sesquiterpenes (15 carbon-based units), presenting mono-, bi-, tri-cyclic and acyclic structural compounds (Fig. 5).^37,40^ On the other hand, aromatic compounds are present in EOs in smaller portions, compared to terpenes. Other compounds identified in EOs include nonvolatile compounds derived from fatty acids and/or glycosides of volatiles, such as jasmonic acid and linalool glucoside, respectively.^41^

### 3.3. EOs for cancer treatment and prevention

A wide variety of health conditions and disorders were treated with EOs that were prescribed as an essential part of traditional/alternative medicine worldwide. This is attributed to the documented therapeutic and medicinal properties of EOs that have further been established by their numerous biological properties acting as antimicrobial, antioxidant, anticancer, antidiabetic, antimutagenic, antibacterial, antiviral, antiprotozoal, anti-inflammatory, and antifungal agents.^97–102^

EOs demonstrated impressive anti-tumor activity against a wide range of cancers, including colorectal, lung, leukemia, gastric, breast, and brain cancers (Table 2).^121–123^ Several mechanisms have been identified for the preventive and anticancer activities of EOs, including antimutagenic, antiproliferative, and antioxidant mechanisms.

**Table 2:** EOs with promising anticancer activities

EOs	Tumor tissue(s)	Mechanism of activity	Ref.
Conifer oil, *Tetraclinis articulate*	Breast cancer, melanoma, and ovarian cancer	Apoptosis induction	103
Cardamom, *Elettaria cardamomum*	Leukemia	Apoptosis induction	104 and 105
Eucalyptus, *Eucalyptus globulus*			
Bayberry/myrtle, *Myrica gale*	Colon and lung cancers	Direct cytotoxic activity against lung carcinoma (A-549 cell line) and colon adenocarcinoma (DLD-1 cell line)	106
Olive oil, *Olea europaea*	Colorectal cancer	Apoptosis induction	107
		Antioxidant activity	
*Foeniculum vulgare*	Liver and breast cancers	Cancer cell line growth inhibition	108
*Eugenia caryophyllata*	Human promyelocytic leukemia	Proliferation inhibition	109
		Apoptosis induction	
		Antioxidant activity	
Galangal, *Alpinia officinarum.* Khus*, Vetiveria zizanioides.* Citronella grass, *Cymbopogon nardus.* Thai lime, *Citrus hystrix.* Beetle leaf, *Piper betle*. *Piper nigrum*. Turmeric, *Curcuma longa*. *Mentha spicata*. *Zingiber montanum*. *Ocimum basilicum*. Palmarosa, *Cymbopogon martini*. *Ocimum americanum*. Grapefruit tree, *Citrus paradise*. *Lavandula angustifolia*. *Ocimum sanctum*	Human mouth epidermal carcinoma and leukemia	Proliferation inhibition	110–113
*Nigella sativa*	Colon cancer and hepatotoxicity	Proliferation inhibition	114 and 115
		Antioxidant activity	
*Matricaria chamomilla*	Glioma	Apoptosis induction	116
*Artemisia annua*	Hepatocarcinoma	Apoptosis induction	117
*Myristica fragrans*	Human neuroblastoma	Apoptosis induction	118
*Pistacia lentiscus* var. *chia*	Colon cancer	Proliferation inhibition	119
	Lewis lung carcinoma	Apoptosis induction	38
		Angiogenesis inhibition	
	Leukemia	Apoptosis induction	120
		Proliferation inhibition	
		Angiogenesis inhibition	

#### 3.3.1. Antimutagenic activities of EOs

The antimutagenic activity exerted by EOs has a key role in cancer prevention, where tumor development can be suppressed *via* several mechanisms. These include enzymatic detoxification of mutagens, direct inhibition of mutagen penetration into cellular membranes, pro-mutagen conversion suppression, mutagen inactivation *via* direct scavenging, error-free DNA restoration, and free radical scavenging.^13^

#### 3.3.2. Antiproliferative activities of EOs

The antiproliferative activity shown by EOs has an essential role in cancer management and can further be established *via* several mechanisms, all of which can lead to disrupting the cell membrane and the death of tumor cells, including apoptosis induction, membrane permeability increase, cellular membrane depolarization, and membrane-bound-enzyme' activity suppression.^14^

#### 3.3.3. Antioxidant properties of EOs

Oxidative stress is an essential characteristic and condition present in tumor microenvironments, where multiple ROS can accumulate due to the inhibition of the electron transport chain. ROS accumulation can induce severe biological damage of proteins, lipids, and nucleotides (*e.g.*, DNA). Major classes of ROS include peroxides (*e.g*., hydrogen peroxides), free radicals (*e.g*., hydroxyl radicals), and superoxide anions.^15,16^ EOs have exhibited major activities in preventing oxidative stress and thus fighting several cancers *via* two common approaches. First, EOs can bind to ROS inside the tumor microenvironment which leads to the activation of a series of events started by phenoxy radical formation. The latter can then bind to ROS present to prohibit any consequent oxidative damage.^15,16^ Eugenol, extracted from clove oil, is a typical example of this mechanism approach.^13^ For the second approach, EOs can initiate the upregulation of several non-antioxidant enzymes (*e.g.*, glutathione) and antioxidant enzymes (*e.g*., superoxide dismutase, glutathione peroxidase, and catalase). The upregulation of such enzymes has a critical role in fighting the oxidative stress associated with tumor development.^17^ Carvacrol and *trans*-caryophyllene in addition to other EOs, extracted from *Wedelia chinensis*, have shown great potential against mice lung cancer *via* augmenting the antioxidant activities of several enzymes including oxidized glutathione.^13,17^

### 3.4. Limitations and challenges of EOs

**Fig. 6:**
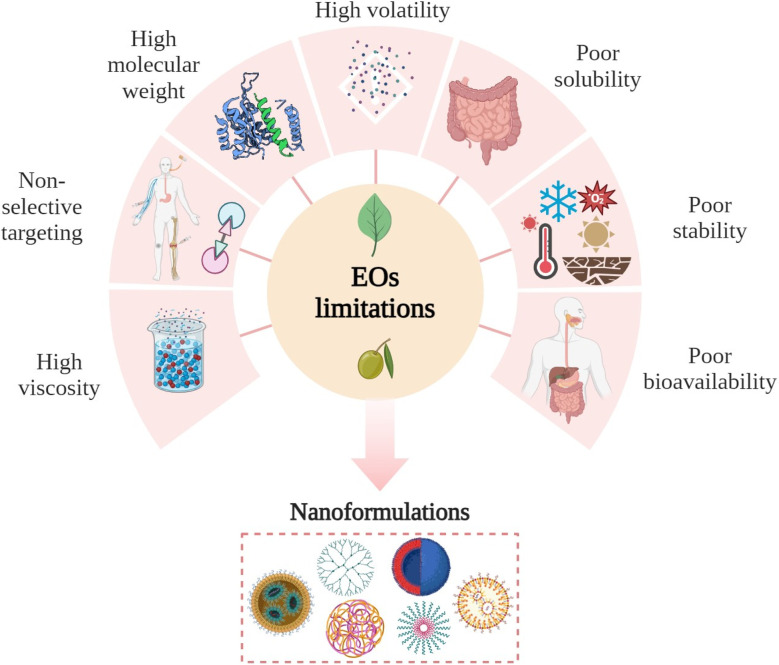
Main limitations associated with the use of EOs for clinical and biological applications. Figure drawn using Biorender.

Despite their impressive biological activities, EOs face challenges limiting their full potential in clinical applications. These challenges can briefly be summarized by the following disadvantageous characteristics such as poor bioavailability, chemical instability, high volatility, and light sensitivity (Fig. 6). Hence, it is essential for this research field to investigate new strategies to deliver and apply EOs, especially in cancer treatment. This focus on improved delivery methods aims to maximize the potential therapeutic benefits of EOs.^18^

## 4. Nanoparticulate-based drug delivery systems for cancer treatment

**Fig. 7:**
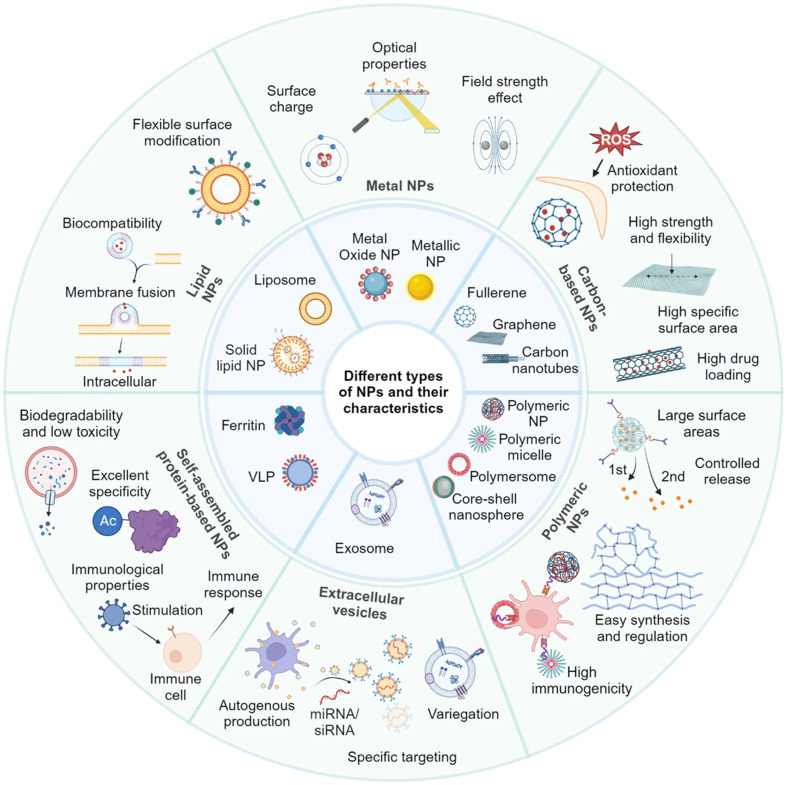
Different examples of nanomaterials classified according to their characteristics and functionality. Reprinted from ref. 124. Copyright 2024 Springer Nature.

The outstanding physicochemical properties of nanomaterials can mainly be attributed to their size at the nanoscale, offering higher active surface areas, which has impacted their optical, chemical, biological, mechanical, and electrical properties (Fig. 7).^125,126^ Hence, nanomaterials might be subcategorized into various groups according to their morphology, size, functionality, state, and characteristics.^127–131^

**Fig. 8:**
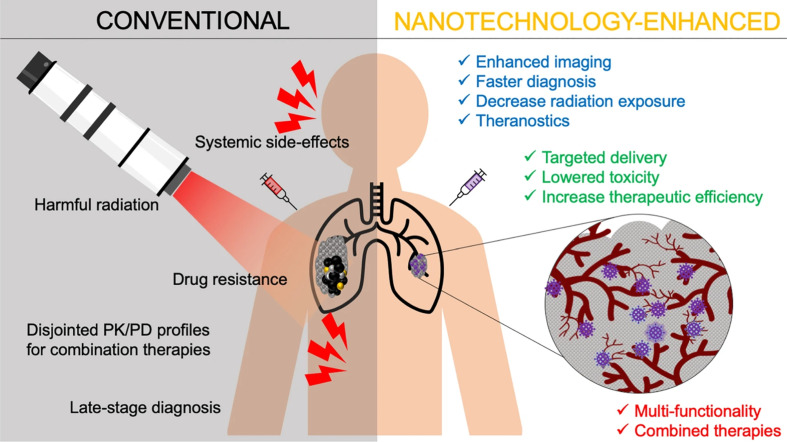
Several advantages achieved by applying nanotechnology for cancer management, compared to conventional radiational therapy, imaging, diagnosis, and anticancer drugs. Lower systemic toxicity and greater therapeutic efficacy of nanomedicines have been well-established at their target site. Nevertheless, several nanomaterials have shown exceptional intrinsic properties, due to their unique chemical and physical properties, which further allowed early cancer diagnosis facilitation, multi-model treatment enhancement (theranostic), radiational therapy localization, multiple drug resistance circumvention, and bioimaging improvement.^132^ (PK = pharmacokinetics and PD = pharmacodynamics). Reproduced from ref. 132. Copyright 2021 Springer Nature.

Drug delivery systems fabricated and based on nanoformulations have been provided with superior characteristics. These characteristic features include prolonged drug release and protection, higher drug permeability against tumor cells, improved drug bioavailability, simultaneous drug loading capability, functionalization possibilities, and enhanced therapeutic potential.^27^ These characteristics tremendously benefited many drugs, natural extracts, bio-entities, and small molecules, improving their delivery and clinical applications as well as overcoming their well-acknowledged limitations, some of which have been mentioned earlier.^20–27,30^ Ultimately, beyond the inherent therapeutic properties of some nanomaterials, their nanoscale features offer exciting possibilities in drug delivery. These features have become well-established tools for enhancing the effectiveness of existing therapies and enhancing their therapeutic efficacies for different clinical applications, particularly for cancer targeting and treatment.^27^ These possibilities and benefits include, but are not limited to, minimizing systemic toxicity and adverse events, augmenting targeting ability, improving pharmacological and pharmaco-dynamic/kinetic profiles, subsiding toxicities associated with chemo- and radiotherapies, and enhancing permeation and localization capabilities of loaded drugs/cargoes (Fig. 8). In addition, improving imaging and diagnostic sensitivity has been widely reported with the use of multiple nanosystems.^27,132^

Moreover, nanoformulations have the ability to eradicate a wide variety of tumors and overcome their resistance mechanisms while augmenting the localization, specificity, accumulation, and stimulating abilities of their encapsulated cargo(s).^133,134^ Various nanocarriers utilize the poor lymphatic drainage and leaky blood vessels surrounding cancer tissues to deliver and bypass the tumor endothelium and passively accumulate inside.^135^ Additionally, other nanocarriers could offer better patient prognosis coupled with an early detection and monitoring of some tumors. This could result in enhancing surgical guidance in tumor resection, surveillance, and treatment monitoring. Therefore, nanomaterials were extensively exploited to manage several malignancies in terms of their treatments, diagnosis, imaging, and therapeutic applications.^136^

**Fig. 9:**
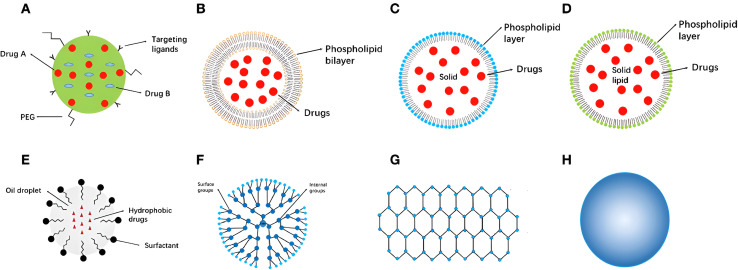
Examples of several nanocarriers utilized for cancer therapy and their structural and/or surface functionalization capabilities. (A): A nanoparticle coloaded with two different drugs and functionalized by attaching certain targeting ligands to its surface. (B): Liposomes structure (the core and the phospholipid bilayer enable the encapsulation of both hydrophobic and hydrophilic drugs simultaneously). (C): Solid lipid nanoparticle structure (a layer of phospholipid and a core inside). (D): Nanostructured lipid carriers a unique structure relatively similar to the one present in solid-lipid nanoparticles. (E): Nanoemulsions with their basic structural elements and loading-functionality (hydrophobic drugs can be encapsulated inside). (F): Dendrimer structure with a wide variety of similar and dissimilar group attachments either on the surface or in-between their structural branches. (G): Graphene structure representing the basic element of a carbon-based nanostructure. (H): Metallic nanoparticles (*e.g.*, gold, silver, and magnetic nanoparticles).^137^ Reproduced from ref. 137. Copyright 2022 Frontiers.

Consequently, several nanocarriers have also shown remarkable targeting abilities due to the unconditional functionalization capabilities of nanocarriers which could enhance their targeting abilities against tumorous tissues.^137–139^ Moreover, several categories of nanomaterials have been utilized for cancer therapy, including lipid-based nanocarriers (*e.g.*, nanostructured lipid carriers, solid lipid nanoparticles, and liposomes), polymeric-based nanocarriers (*e.g.*, dendrimers, micelles, and nanofibers), metallic-based nanocarriers (*e.g.*, magnetic and gold nanoparticles), and carbon-based nanocarriers (*e.g.*, multi-wall carbon nanotubes and graphene)^137^ (Fig. 9).

### 4.1. Nanocarriers utilized for drug delivery against cancer

#### 4.1.1. Lipid based nanoparticles

**Fig. 10:**
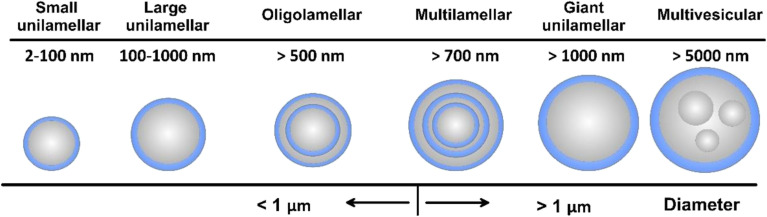
Types and size ranges of liposomes.^140^ Reproduced from ref. 140. Copyright 2022 MDPI.

**Fig. 11:**
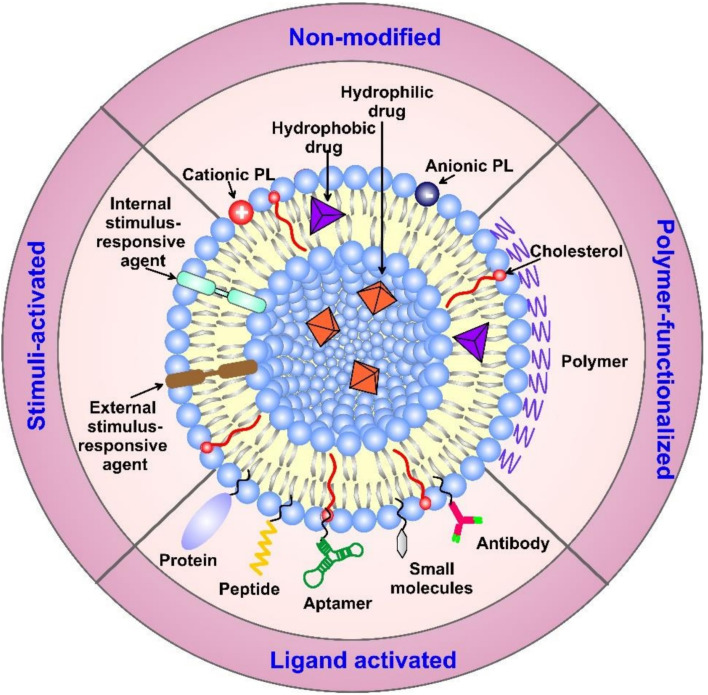
Liposome's structure, surface functionalization, and classification (based on the modifications applied). Surface functionalization can vary with the sort of ligands attached. Additionally, liposomes can carry various biologically active components and targeting moieties such as proteins, hydrophobic and hydrophilic drugs, antibodies, aptamers, peptides, and nucleotides inside their structure and/or on their surface. The targeting moieties can provide liposomes with additional properties, *i.e.*, the ability to release their cargo(s) in response to different stimuli.^140^ (PL = phospholipid). Reproduced from ref. 140. Copyright 2022 MDPI.

Liposomes can be prepared in different sizes (2 to 5000 nm) and their structure comprises a hydrophilic core surrounded by a lipid bilayer, where the lipid bilayer can be composed of cholesterol and one or multiple phospholipidic layers (Fig. 10). Thus, both hydrophilic and hydrophobic moieties can be encapsulated. Several types of liposomes have been developed, including small unilamellar, large unilamellar, multilamellar, and multivesicular liposomes (Fig. 10). Additionally, liposomes are distinguished from other nanocarriers by their favorable biocompatibility, owing to the propitious structure they hold which is very similar to the one present in biological cell membranes. Moreover, liposomes offer a functional surface with a wide variety of modifications and functionalizations (Fig. 11). Hence, different benefits are associated with the use of liposomes as nanocarriers such as enhancing bioavailability, sustainability, solubility, and targeting capability of the loaded drugs.^141^

Niosomes can be defined as micro-/nano-vesicles primarily composed of hydrated nonionic surfactants and cholesterol, or cholesterol derivatives.^142^ Notably, niosomes have the advantageous capability of encapsulating a diverse range of substances, encompassing both hydrophilic and lipophilic active biomolecules.^24,142^

#### 4.1.2. Polymeric nanoparticulate systems

**Fig. 12:**
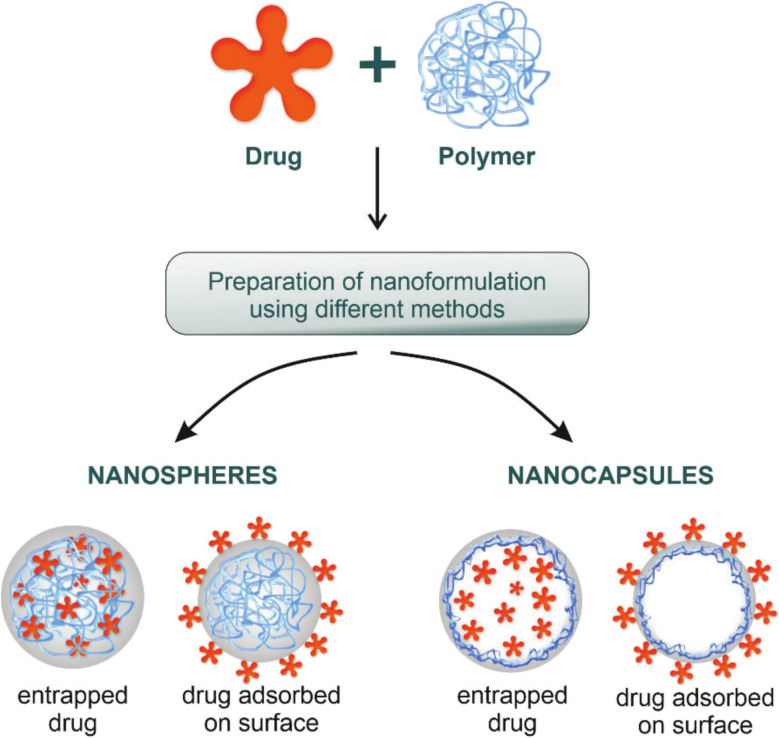
Nanocapsules and nanospheres are two common polymeric-based nanoparticles utilized for different bio-applications, including drug delivery. The unique structure in which they can carry the drug of interest can differ, where nanocapsules are able to entrap the drug inside their core which is surrounded by a layer of polymer. In contrast, nanospheres have the ability to entrap the drug of interest inside their cross-linked polymeric core (the core is formed of a cross-linked polymer). Aside from the core part, both nanosystems could adsorb drugs on their surfaces.^145^ Reproduced from ref. 145. Copyright 2022 MDPI.

Polymeric nanoparticles can have a size range between 1 nm and 1000 nm. For polymeric nanoparticles, nanospheres and nanocapsules have commonly been developed. In the case of nanocapsules, a drug of interest can be encapsulated inside a core and further be enclosed by a layer of polymer. However, when a polymer is cross-linked inside the core of the structure then the developed system can be called nanospheres, where the drug of interest is loaded inside the cross-linked polymer. Also, both nanocapsules and nanospheres could adsorb the drug of interest on their surfaces^143,144^ (Fig. 12).

Moreover, other polymeric-based delivery nanosystems have been developed, where both synthetic polymers (*e.g.*, polylactic acid (PLA), polylactic-*l*-acid (PLLA) and PLGA) and natural polymers (*e.g.*, proteins, cellulose, and starches) have offered a promising platform for the synthesis of several polymeric-based nanoparticles such as micelles, nanofibers, and dendrimers. Various medical and pharmaceutical applications have exploited the outstanding properties, biocompatibility, and biodegradability of polymeric nanoparticles particularly for drug delivery purposes.^141,145–148^ Furthermore, polymeric-based nanoparticulate drug delivery systems have demonstrated favorable characteristics for their cargo(s) such as higher stability, bioavailability, sustainability, and prolonged circulating half-life. Additionally, polymeric nanosystems can be developed based on thermosensitive polymers to eventually have superior release control and targeted delivery against tumor tissues.^141,145,148,149^

**Fig. 13:**
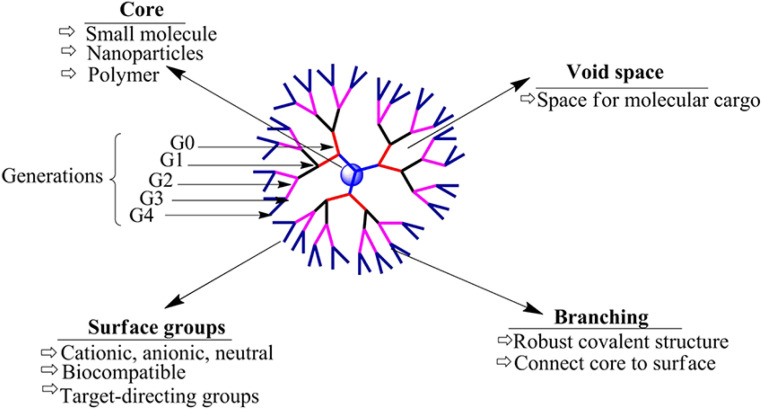
Basic structure of dendrimers, revealing their key structural characteristics.^150^ Reproduced from ref. 150. Copyright 2017 Frontiers.

**Fig. 14:**
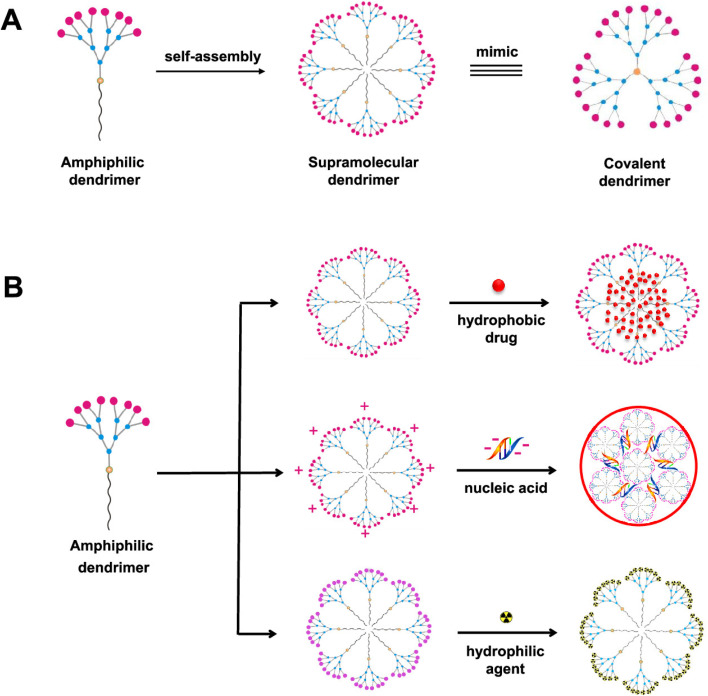
Amphiphilic dendrimers' (A) self-assembly and (B) capability to provide a promising nanocarrier platform for carrying various therapeutics, biomolecules (*e.g.*, nucleotides), hydro-philic/phobic drugs, and other natural or synthetic agents. Small dendrimers can self-assemble into supramolecular dendrimers, similar to covalent dendrimer structures, revealing the ability to develop more stable structures that can be utilized for different bio-applications which is particularly advantageous for drug delivery purposes.^151^ Reproduced from ref. 151. Copyright 2020 ACS.

Dendrimers refer to a group of polymeric macromolecules with a size that ranges from 1 nm to 100 nm. Additionally, dendrimers have globular shapes and yet sophisticated spherical structures. The general structure of dendrimers is composed of four main elements, (1) a core which can be a nanoparticle, polymer, or any small biomolecule, (2) branches which connect the core to the surface which can also be called generations, depending on the number of branches developed, (3) repeated units with one or more branch junction(s), and (4) functional surface groups of different natures, sizes, charges, bioactive properties, targeting functionalities, *etc.*^141,150,151^ (Fig. 13). The surface group variability and modifiability provide advantageous characteristics for dendrimers for targeted delivery and for protecting their loaded or coloaded drugs. Thus, such controllable architecture present in dendrimers allows the attachment of various biomolecules to enhance the passive entrapment and the release of their cargo(s). On the other hand, dendrimers can self-assemble and further form more stabilized nanostructures. Furthermore, dendrimers might be joined with carbon nanotubes, nanoparticles, or liposomes to develop a greater nanocarrier (*i.e.*, developing inorganic or organic hybrid nanoparticles), exploiting the modifiability offered by dendrimers. The obtained nanocarriers can show superior bioavailability and targeting capabilities (*e.g.*, loading anticancer agents, imaging and detecting agents, radioligands, and affinity ligands).^141,150,151^ (Fig. 14).

**Fig. 15:**
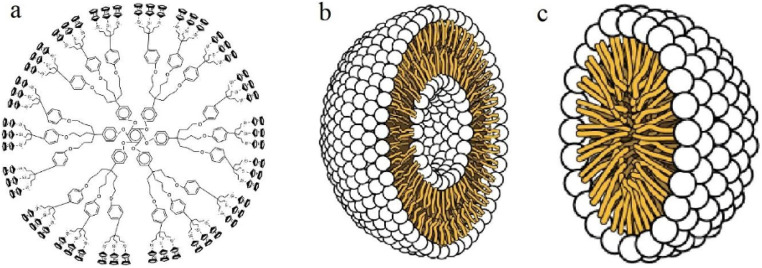
Basic structures of three groups of promising nanocarriers utilized for different bio-applications, particularly for cancer targeting, (a) dendrimers, (b) liposomes, and (c) micellar nanoparticles.^152^ Reproduced from ref. 152. Copyright 2017 IOPscience.

Micellar nanoparticles characterize another promising group of spherical organic nanocarriers comprising a simple structure of a core that is surrounded by a shell (Fig. 15). Micellar nanoparticles could be obtained in an aqueous medium with the self-assembly of amphiphilic block copolymers. In contrast to liposomes, the amphiphilic part is arranged in a monolayer surrounding a hydrophobic core which further composes a hydrophilic surface and hydrophobic tails.^141,152,153^ Moreover, the shell can usually be presented by hydrophilic polymers (regularly PEG); additionally, other hydrophobic polymers have been used to form the shell (*e.g.*, PCL, PVP, PLGA, and PLA), establishing micelles with superior biodegradability, bioavailability, and biocompatibility. Also, the core can entrap hydrophobic drugs, serving as a reservoir to maintain several natural compounds and drugs. Hence, a wide variety of chemotherapeutics and natural compounds have shown greater retention effect and permeability owing to the higher solubility, targeting ability, sustainability, circulation half-life, and bioavailability provided by these nanocarriers.^141,152,153^

#### 4.1.3. Nanoemulsions

**Fig. 16:**
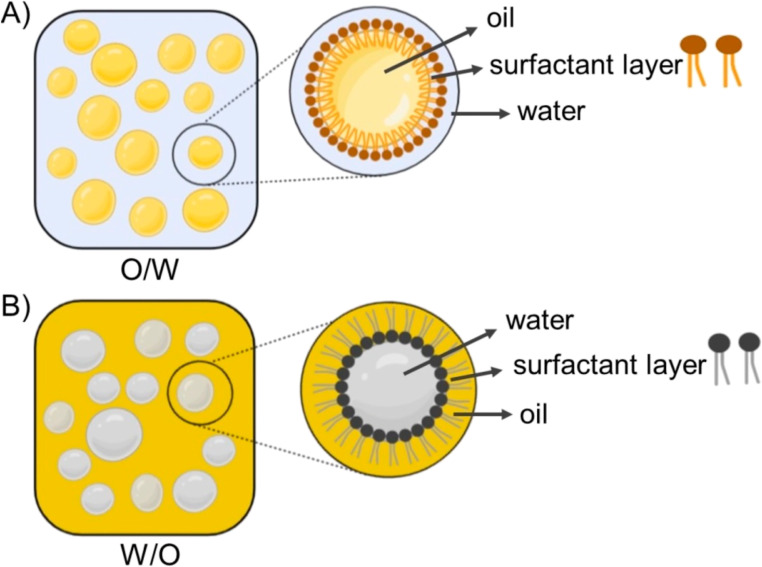
Schematic representation of the two common types of nanoemulsions utilized. (A) The oil in water nanoemulsion type and (B) the water in oil nanoemulsion type.^154^ (O/W: oil in water; W/O: water in oil). Reproduced from ref. 154. Copyright 2022 MDPI.

Nanoemulsions are formed by dispersing two immiscible liquids in each other to eventually develop a heterogeneous system in which the emulsified particles have a size less than 1000 nm (Fig. 16). While conventional emulsions have a milky appearance, nanoemulsions have a rather translucent appearance.^18,154–158^ More importantly, nanoemulsions have been broadly utilized for drug delivery and other bio-applications, owing to the remarkable advantages they afford such as larger surface area and smaller sizes, augmented solubility and bioavailability, improved sustainability, increased dispersibility of hydrophobic drugs, and greater stability.^18,154–158^

#### 4.1.4. Solid lipid nanoparticles

**Fig. 17:**
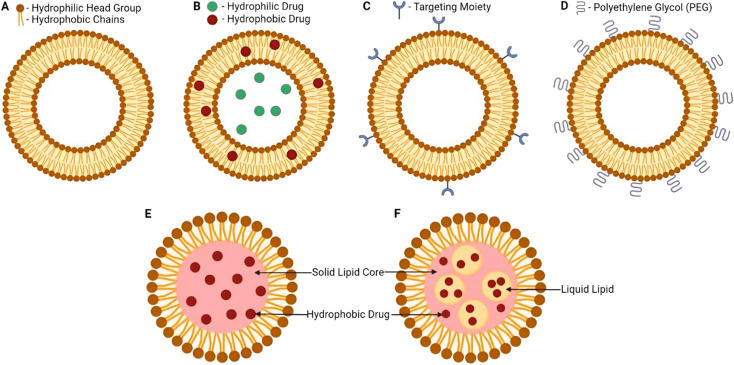
Lipid-based nanocarrier structural comparison. (A) Basic structure of liposomes, (B) liposome nanocarrier loaded with both hydrophilic and hydrophobic drugs in the core and the phospholipid bilayer of the liposome, (C) liposome nanocarrier with a surface modification of a targeted moiety attached to increase targeting, (D) liposome nanocarrier with several PEG moieties attached to the surface, (E) basic solid lipid nanocarrier structure revealing a solid lipid core involved, and (F) basic nanostructured lipid carrier structure revealing a binary system entailed both solid and liquid lipidic cores forming a basic biocompatible matrix.^154^ Reproduced from ref. 154. Copyright 2022 MDPI.

Solid lipid nanoparticles have recently attracted increasing interest in the drug delivery field, where they have been utilized for the loading and delivery of peptides, drugs, cosmetics, proteins, nucleotides, and nutraceuticals. Solid lipid nanoparticles can be defined as a group of organic nanoparticles prepared in the form of dispersions which is very similar to the oil in water nanoemulsion except that the liquid lipid phase had been replaced by a solid lipid phase, at ambient temperature (Fig. 17). As a result, such nanoformulations could promisingly entrap several drugs and active metabolites.^154,159,160^

#### 4.1.5. Nanostructured lipid carriers

Nanostructured lipid carriers can be identified as the second generation of solid lipid nanocarriers, where larger drug amounts can be encapsulated, lower water content can be incorporated, reduced microbial growth and minimized leakage could be achieved, and more importantly greater improvements in drug entrapment, stability, and bioavailability could be attained. Such advantages could only be attributed to the binary system afforded by the nanostructured lipid carriers in which both liquid and solid lipids are present. Hence, these carriers could be promisingly utilized for drug delivery applications due to the higher biocompatibility, biodegradability, stability, and bioavailability guaranteed for their loaded drugs^154,161,162^ (Fig. 17).

#### 4.1.6. Nanofibers

**Fig. 18:**
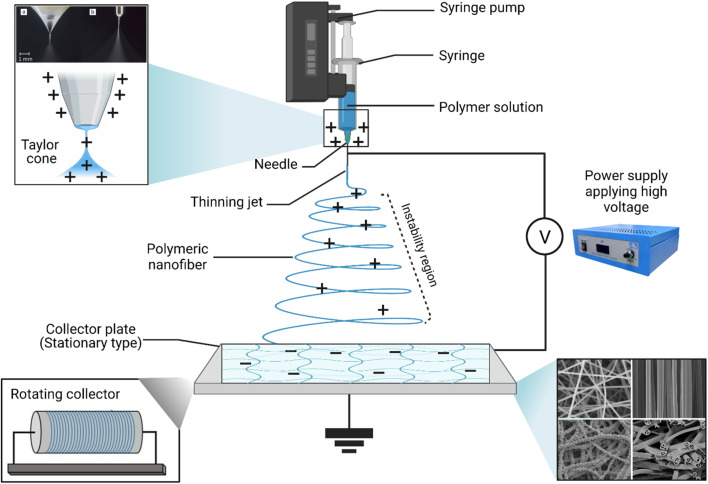
Schematic representation of the conventional electrospinning device and its basic components. In addition, the electrospinner can have two common set-ups, a vertical (shown here) and a horizontal set-up, where the collector plate can be placed in a horizontal line facing a needle on the other side. Furthermore, the figure reveals the Taylor cone that is formed during the electrospinning process while applying a high voltage to the needle (usually metallic), thus initiating the nanofiber formation from the polymeric solution. The opposite charges that are offered from the collector plate help collect and grab the nanofibers on the plate surface. Figure drawn using Biorender.

Nanofibers refer to a group of nanomaterials that could be obtained in a fiber form with a diameter that ranges from tens to hundreds of nanometers.^163^ Many techniques could be used for nanofiber production, including electrospinning, phase separation, template synthesis, and self-assembly.^164–166^ Nonetheless, electrospinning, which is based on electrostatic driving forces, can be considered the prime method exploited to obtain nanofibers having incomparable properties for encapsulating numerous chemotherapeutics, EOs, and other natural extracts and biomolecules (Fig. 18).^167^ This might be explained by the rapid, simple, and cost-effective characteristics that distinguish the electrospinning process.^168^ Furthermore, electrospun nanofibers have superior advantages while utilizing a wide variety of natural, synthetic, and hybrid polymers to obtain nanofibers with a diameter range of a few nanometers and a large surface area. Also, electrospun nanofibers could exhibit favorable mechanical strength properties, ease of functionalization and scaling-up feasibility, high interstitial spaces, and exceptional physical and chemical properties.^163,169^ The encapsulation of natural extracts, including EOs, into nanofibers could encourage the development of innovative treatment approaches for targeted delivery, tissue engineering (scaffold development), and wound dressing and healing (including diabetic wounds). Besides, the encapsulation of EOs has increased their targeting ability, sustainability, bioavailability, stability, and therapeutic potential while minimizing their limitations.^169,170^

**Fig. 19:**
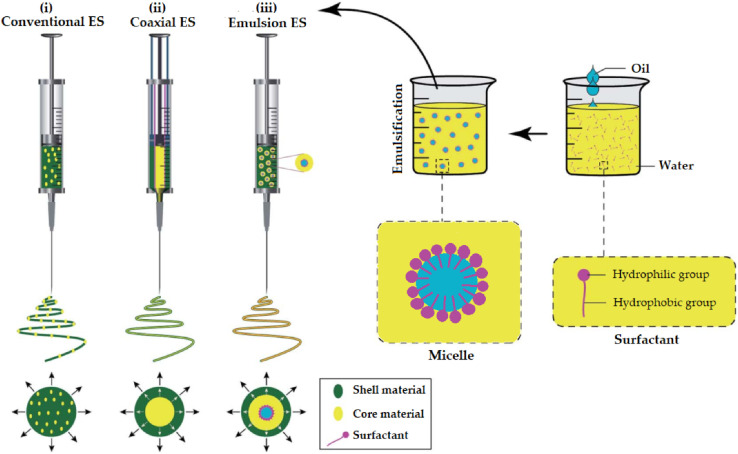
Schematic representation of (i) a spinneret utilized for conventional electrospinning, (ii) a spinneret used for coaxial electrospinning, where two feeding systems are electrospun synchronously, and (iii) a spinneret utilized for emulsion electrospinning, where the major difference is that in this method two immiscible liquids can be electrospun simultaneously. (ES = electrospinning).^171^ Reproduced from ref. 171. Copyright 2022 MDPI.

Promising advantages have been associated with the use of nanofibers obtained by either conventional (Fig. 18) or coaxial (Fig. 19) electrospinning methods for loading EOs, compared to the conventional polymeric films, including^169,171–173^

• Higher protection against degradation, decomposition, heat, light, and other chemical and environmental conditions that might affect the stability, and the overall therapeutic efficacy of the EOs loaded.

• Improved targeted and sustainable release profiles.

• Superior mechanical properties offered which could consequently support the oily and elastic nature of the EOs loaded (*e.g.*, compression).

• Numerous medicinal, pharmaceutical, food packaging, biological, and drug delivery applications have exploited the exceptional properties of the scaffolds obtained *via* electrospinning, in which such scaffolds have shown remarkable biodegradable and biocompatible properties.

### 4.2. Nanocarrier mechanisms to target tumor tissues

Tumor tissues have several characteristics including angiogenesis, inflammatory marker eruption, receptor upregulation, and other physiologic changes that exploit and predominate the microenvironment surrounding the tumor tissues. As a result, nanoparticles and other nanomaterials could exploit similar abnormal changes developed inside and around the tumor microenvironment to deliver their cargo(s) *via* two common mechanisms which are passive and active mechanisms.^145^

#### 4.2.1. Passive targeting

**Fig. 20:**
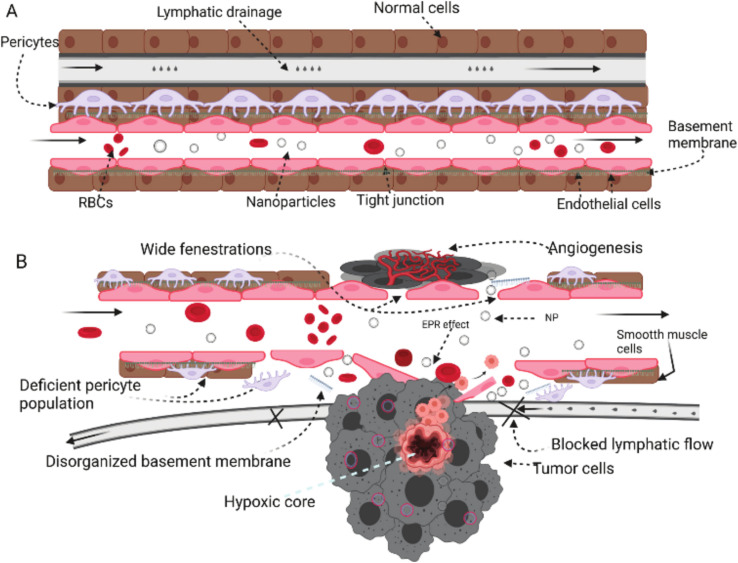
Comparison between normal tissue (A) and tumor tissue (B) while being targeted by nanoparticles. In normal tissues, the absence of an open door for nanoparticles prevents their permeation and/or accumulation inside the cells due to the intact cell membrane, tight junctions, and normal lymphatic drainage. In contrast, tumor cells can invade and disrupt the normal physiologic environment of the normal tissues, establishing their host microenvironment, which leads to a series of abnormal events including cell membrane disruption, hypoxic tissue development, angiogenesis initiation, permeation enhancement, and lymphatic drainage increase. These series of events forming the hypoxic microenvironment surrounding tumor tissues could be exploited by nanoparticles to deliver their cargo(s) efficiently and passively while taking advantage of the higher permeability and prolonged retention effect provided inside the tumor microenvironment.^145^ Reproduced from ref. 145. Copyright 2022 MDPI.

**Fig. 21:**
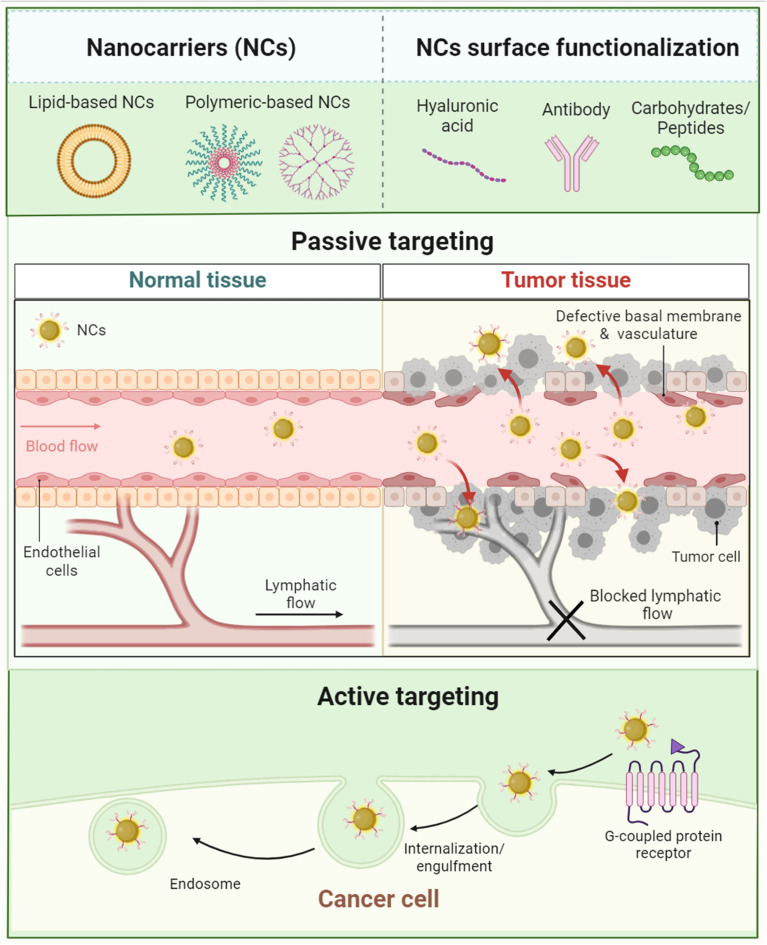
Comparison between the passive and active mechanisms exploited for cancer targeting by nanoparticles. Figure drawn using Biorender.

Among the abnormal physiological changes that surround the tumor microenvironment is the eruption of several cell types including different immune cells such as tumor associated macrophages and fibroblast-associated cells. Furthermore, the development of new blood vessels (angiogenesis), a key characteristic associated with tumor development, is the main reason behind the higher proliferation and cellular influx into tumor environments. These characteristics are essential for tumor survival, providing oxygen and other nutrients; hence, angiogenesis occurs in an irresistible and fast-paced manner. Therefore, these changes allow a higher permeation of several nanoparticles, efficiently and passively, exploiting the abnormalities and great permeability originated in the blood vessels' endothelium. Additionally, the anomalous vascular structure of the lymphatic system could further permit a protracted retention (*i.e.*, a prolonged retention effect) for the nanoparticles inside the tumor tissue due to the greater lymphatic drainage developed.^145,174–177^ Eventually, the greater permeability and prolonged retention effect might be the key characteristics of the passive mechanism exploited by nanoparticles to disrupt, target, and deliver their cargo(s) inside cancer tissues (Fig. 20 and 21).^145,174–177^ Genexol PM (paclitaxel loaded) is a typical example of a clinically approved polymeric-nanoformulated anticancer drug which exploits the passive mechanism approach against metastatic breast cancer.^145,176^

#### 4.2.2. Active targeting

This mechanism mainly depends on the receptors overexpressed on the tumor cell surfaces. While the tumor progresses, the upregulation of specific receptors on the tumor cell surfaces can be observed. Hence, several nanoparticles exploit such a feature whilst being functionalized by the attachment of certain complementary ligands to augment their targeting towards cancer cells. Eventually, the specific ligands that are attached to the nanoparticles can target their complementary receptors expressed on the tumor cell surfaces, allowing better targeting and lower systemic toxicity of their loaded drug(s) (Fig. 21).^178–181^ For instance, several ligands were attached to facilitate this targeting strategy, aiming to target specific-cellular receptors, proteins, and/or antigens, such as PEG, folic acid, aptamers, biotin, antibodies, fluorescent dyes, and carbohydrates (*e.g.*, dextran).^178–181^ These ligands have been commonly attached to liposomes and polymeric nanoparticles for cancer targeting systems and even for circumventing the blood–brain barrier.^178–181^ Nevertheless, ligand-attached nanoparticles have been reported with a higher uptake by the phagocytic system inside biological systems, which negatively affects their circulation half-life. Additionally, ligand-attached nanoparticles might face another challenge in which the cancer stage can determine and impact the variability of the receptors expressed on the surface of the cancer cells, affecting the specificity of the ligands attached to the nanoparticles exploited. Eventually, the higher uptake by phagocytic biosystems and the cancer cell receptor variability represent the main challenges of the active mechanism approach. However, the active mechanism has been more frequently exploited compared to the passive mechanism for cancer targeting.^178–181^ A typical example of a nanoformulated system that exploits the active mechanism is the one developed by Shi *et. al.* (2015) by attaching VEGF to polylactic-*co*-glycolic acid (PLGA) nanoparticles, loaded with paclitaxel, to target human umbilical vein endothelial cells.^182^ Indeed, the developed nanosystem revealed higher affinity and antiproliferative activity compared to free paclitaxel.^182^

## 5. Nanosystems loaded with EOs for cancer therapy

**Fig. 22:**
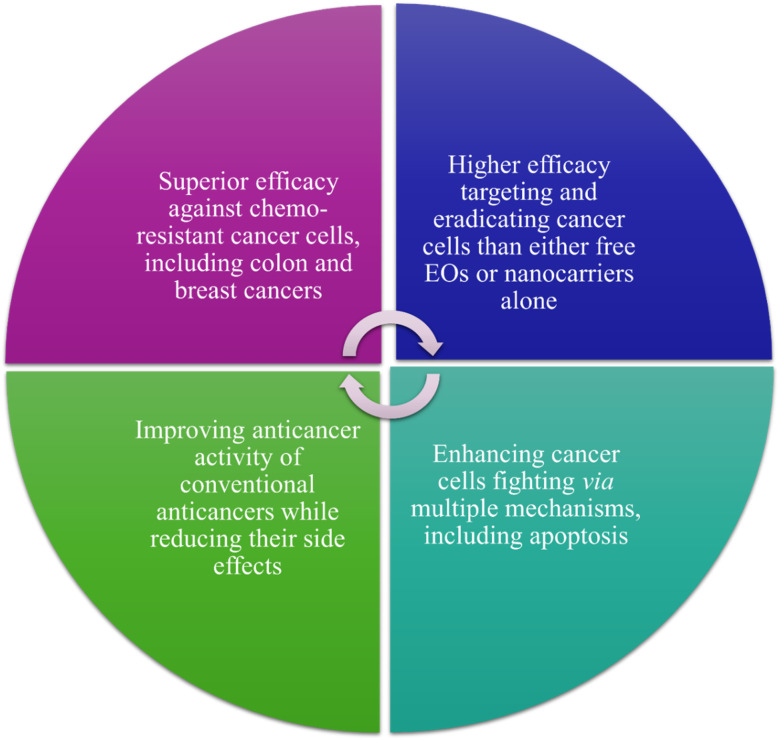
Main advantages obtained while exploiting different nanoformulations for carrying EOs in cancer therapy in comparison to free EOs.

The numerous advantages of nanomaterials, which have been discussed earlier, have encouraged their use for the delivery of different essential oils for cancer targeting. Various nanoparticles, nanoemulsions, nanofibers, nanofilms and composites, and other nanoformulations have been utilized for encapsulating essential oils to improve their encapsulation capacity, stability, biocompatibility, targeting, sustainability, and bioavailability, while reducing their off-target activity and side effects. More importantly, such nanoformulations would protect their encapsulated or loaded essential oils from environmental conditions that are known to negatively influence their activity such as chemical oxidization, temperature and light degradation, and high volatility^18^ (Fig. 22).

**Fig. 23:**
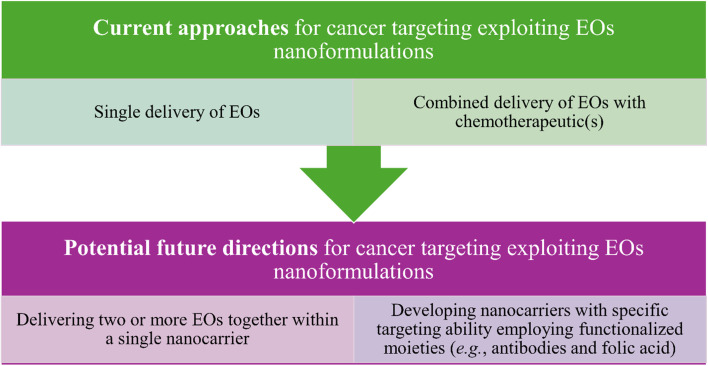
Current and possible future strategies that have been investigated and proposed to improve the anticancer activity of the nanoformulations utilized for carrying EOs.

In addition to the exceptional functionalization properties, nanocarriers can provide a larger surface to volume ratio owing to their unique sizes which consequently can optimize their reactivity, an advantage which is of special interest while targeting tumor tissues inside biological systems. Common nanocarriers utilized for the delivery of essential oils are usually based on lipid and polymeric materials or even a combination of both.^183^ Nevertheless, several strategies have recently been proposed to enhance the delivery of essential oils while being carried inside different nanoformulations, compared to the current ones. These strategies have so far been focused on the possibilities to (1) co-deliver at least two essential oils inside a single platform of a nanoformulation and (2) augment the targeting ability against tumorous tissues *via* the attachment of different targeting moieties such as carbohydrates, antibodies, folic acid, other small molecules, *etc.* Thus, such strategies are basically aiming to augment the anticancer activity and reduce the side effects of the nanosystems utilized to load various EOs^18^ (Fig. 23).

### 5.1. Lipid-based nanoparticles

**Fig. 24:**
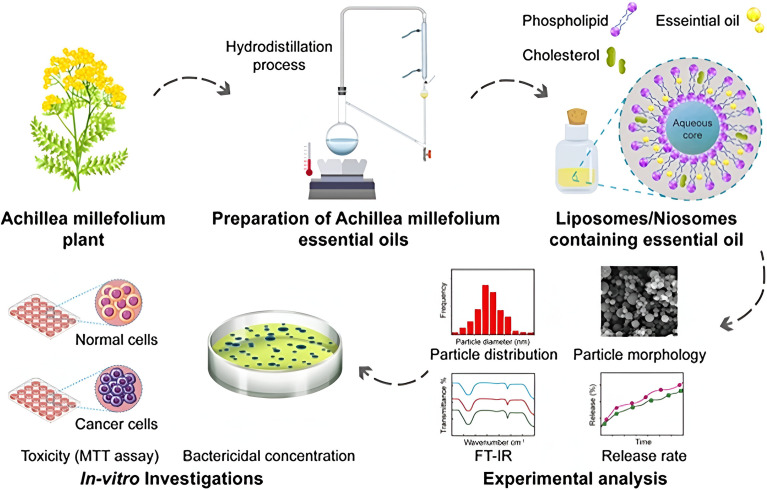
Schematic illustration of nanoencapsulation of *A. millefolium* EOs into liposomes and niosomes to enhance their physicochemical properties, cytotoxic activity, and antimicrobial properties compared to free EOs. Reprinted from ref. 184. Copyright Wiley 2022.

Emtiazi *et al.* (2022) encapsulated the EOs of *Achillea millefolium* into liposomes and niosomes, as depicted in Fig. 24.^184^ The developed nanosystems were compared in terms of their physicochemical characteristics, cytotoxic activities, and antimicrobial properties. The encapsulated liposomes showed an entrapment efficiency (EE%) of 77.5 ± 2.37, size of 196.6 nm, polydispersity index (PDI) of 0.235, and average zeta potential (*Z*-potential) of −47.6 mV indicating a homogeneous system with anionic surface charges of the developed liposomes. On the other hand, the encapsulated niosomes exhibited an EE% of 41.1 ± 2.05, size of 96.8 nm, PDI of 0.318, and *Z* potential of −0.9 mV indicating a homogeneous system with anionic surface charges of the niosomes.^184^ Additionally, the released amount of EOs from the niosomes reached 34.7% compared to 48.8% released from the liposomes after 72 h at pH 7.4 and 37 °C, referring to an enhanced release profile of the encapsulated EOs achieved particularly by the niosome nanoparticles.^184^ More importantly, while both nanosystems showed greater cytotoxic activity against MCF-7 breast cancer cells compared to the free EOs of *A. millefolium*, the encapsulated niosomes showed almost twice higher cytotoxic activity than the encapsulated liposomes, where a 50% reduction in MCF-7 cell viability was reported with the cells treated with 125 μg mL^−1^ of encapsulated niosomes compared to 250 μg mL^−1^ of encapsulated liposomes.^184^ Also, an 80% reduction in MCF-7 cell viability was achieved when cells were treated with 250 μg mL^−1^ of encapsulated niosomes. In addition, the safety profile of both nanosystems could be established against normal human fibroblast (HFF) cells, where both encapsulated niosomes and liposomes showed no cytotoxic activity against the treated HFF cells.^184^ These findings refer to the enhanced stability, sustainability, and therapeutic activity of the EOs of *A. millefolium* upon their encapsulation into the lipid-based nanoparticles of niosomes and liposomes. On the other hand, the antimicrobial properties of the encapsulated nanoparticles with *A. millefolium* EOs were investigated by Emtiazi *et al.* (2022) against *E. coli* and *S. aureus* and were compared to the free EOs of *A. millefolium*.^184^ Interestingly, the encapsulated liposomes showed a minimal inhibitory concentration (MIC) of 0.21 mg mL^−1^, minimal bactericidal concentration (MBC) of 0.43 mg mL^−1^, and diameter of the growth inhibition zone of 21 ± 2.29 mm against *E. coli* compared to free EOs of *A. millefolium* which showed an MIC of 0.25 mg mL^−1^, MBC of 0.5 mg mL^−1^, and diameter of the growth inhibition zone of 16 ± 1.3 mm against the same bacterium.^184^ Remarkably, the encapsulated niosomes could exhibit a greater activity against *E. coli* with an MIC of 0.062 mg mL^−1^, MBC of 0.125 mg mL^−1^, and diameter of the growth inhibition zone of 25 ± 0.5 mm.^184^ Furthermore, the antimicrobial activity of encapsulated liposomes against *S. aureus* was revealed with an MIC of 0.21 mg mL^−1^, MBC of 0.43 mg mL^−1^, and diameter of the growth inhibition zone of 19 ± 1.8 mm compared to free EOs of *A. millefolium* which showed an MIC of 0.5 mg mL^−1^, MBC of 1 mg mL^−1^, and diameter of the growth inhibition zone of 13 ± 1.8 mm against *S. aureus*.^184^ Similarly, the encapsulated niosomes could show the highest antibacterial activity against *S. aureus* with an MIC of 0.125 mg mL^−1^, MBC of 0.25 mg mL^−1^, and diameter of the growth inhibition zone of 22 ± 1.73 mm.^184^ Overall, the lipid based nanocarriers used in this study could effectively augment the EOs' physicochemical properties, bioavailability, and antimicrobial activity against common pathogens known to cause serious infectious diseases to humans, and ultimately superior cytotoxic activity against breast cancer cells.

**Fig. 25:**
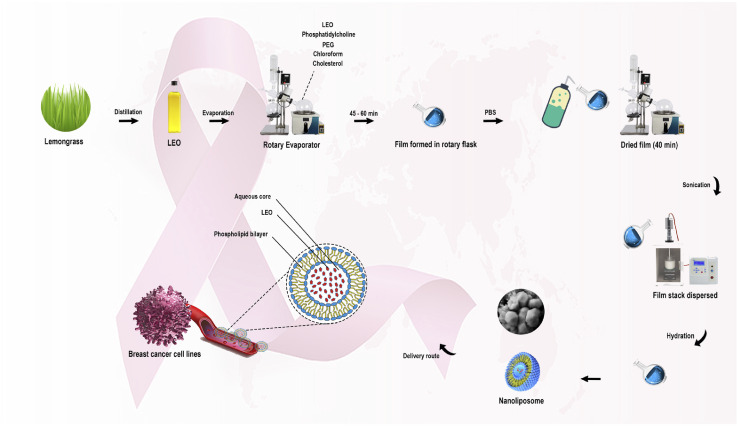
Schematic diagram illustrating the extraction of lemongrass EOs and their encapsulation into nanoliposomes to enhance their anticancer activity against breast cancer cell lines in comparison to free EOs. Reprinted with permission from ref. 185. Copyright Elsevier 2023.

Rahimi *et al.* (2023) reported the encapsulation of lemongrass EOs into nanoliposomes to enhance their anticancer activity against several breast cancer cell lines, as shown in Fig. 25.^185^ The obtained liposomes had a size of 53.97 nm, PDI of 0.273, EE% of 70, and *Z*-potential of −26 ± 0.52 mV indicating a monodispersed system consisting of particles with charged surfaces.^185^ Also, the GC-MS of EOs showed the following major components: geraniol, neral, and geranial.^185^ More significantly, the encapsulated liposomes exhibited higher cytotoxic activity against breast SKBR3, MDA-MB-231, and MCF-7 cancer cells, using a concentration of 100 μg mL^−1^ of lemongrass EOs, where the treated cells with liposomes showed cell death rates of 72.7%, 64.5%, and 66%, respectively.^185^ In contrast, the lemongrass EOs showed lower cytotoxic activity against SKBR3, MDA-MB-231, and MCF-7 with cell death rates of 44%, 32.7%, and 39%, respectively, using the same concentration.^185^ Additionally, the flow cytometric analysis showed an improvement in the apoptosis rate of the lemongrass EOs upon their encapsulation into liposomes by approximately 20% against the treated cancer cells, utilizing the same concentration of 100 μg mL^−1^.^185^ These results depict the effective targeting ability achieved by the encapsulation of the reported EOs into liposomes, revealing such nanoparticles to be promising nanocarriers to combat breast cancer.

The EOs of ginger (*Zingiber officinale*) have shown remarkable antioxidant and antimicrobial activities. Also, the FDA listed ginger as a *Generally Regarded as Safe* (GRS) substance.^186^ Hence, Ekrami *et al.* (2023) could successfully encapsulate the EOs of ginger rhizomes (*Zingiber officinale*) into liposomes with a size of 100 nm, *Z*-potential of −18.17 ± 1.17 mV, PDI of 0.293 ± 0.009, and EE% of 66.24%.^187^ More importantly, the developed liposomes exhibited outstanding antioxidant activity, with 83% radical scavenging activity reported using DPPH assay, and stability. The stability test could be accomplished over 24 h using UV light (254 nm), to investigate the degradation ability of the free EOs compared to the ones encapsulated into liposomes. Remarkably, after 18 h, the free EOs showed 99% degradation compared to approximately 25% shown with those encapsulated into the liposomes.^187^ These findings refer to the greater stability and biological properties of the ginger EOs imparted by encapsulation into liposomes. Furthermore, the cytotoxicity of both free and encapsulated ginger EOs was assessed on human colon cancer (HT-29) and normal human umbilical vein endothelial cells (HUVEC).^187^ Unpredictably, the free EOs showed higher cytotoxic activities against both HUVEC and HT-29 cells after 24 h of incubation using the same concentration of ginger EOs of 222 μg mL^−1^. While both free and encapsulated ginger EOs showed negligible reduction in the viability of the normal cells (HUVEC), their effects on HT-29 cells could significantly differ. The free EOs showed around 75% of reduction in HT-29 cells compared to approximately 20% reduction in HT-29 cells treated with the encapsulated liposomes.^187^ The lower cytotoxic activity of the encapsulated liposomes against the cancer cells (HT-29) could be explained by the slower and sustained release of the ginger EOs from their lipid nanocarriers within a narrow window of time (24 h). Hence, such findings might be interpreted and enforced by extending the MTT assay study to cover an extended period of incubation of the encapsulated nanoparticles with the treated cells (*i.e.*, 72 h incubation).

The EOs of *Origanum vulgare*, which belongs to a mint plant of the Lamiaceae family, have depicted a wide variety of promising antioxidant, anticancer, and antimicrobial activities.^188^ Hence after, Kryeziu *et al.* (2022) reported the encapsulation of *O. vulgare* EOs, containing a major component of carvacrol (71.41%), into liposomes mainly to improve their cytotoxic and antioxidant properties.^189^ The obtained liposomes, encapsulating *O. vulgare* EOs, showed a size range of 89 ± 0.91 to 319 ± 20.5 nm, PDI values within 0.16 ± 0.02 and 0.28 ± 0.01, *Z*-potential between −8.4 ± 0.3 and −26.9 ± 0.9 mV, and EE% between 83.5 ± 3.5 and 85.5 ± 2.4%,^189^ reflecting a well-stable profile of the developed nanocarriers with promising encapsulation efficiencies. Interestingly, the encapsulated liposomes exhibited remarkable improvement in the antioxidant activity of the *O. vulgare* EOs reaching 84.67 ± 1.53% scavenging activity of free radicals, using a DPPH assay, compared to the free EOs which showed a scavenging activity of 53.52 ± 4.27% of free radicals.^189^ Moreover, the encapsulated liposomes showed a significant enhancement in the cytotoxic activity of the *O. vulgare* EOs against breast cancer cells, reducing the cell viability of the MCF-7 cells to 25.89% compared to 50.10% reduction showed in cells treated with free EOs alone, after 24 h incubation and applying the same EO concentration (25 μg m^−1^).^189^ These findings indicate the potential use of similar nanocarriers as promising anticancer agents, encapsulating the EOs of *O. vulgare*.

**Fig. 26:**
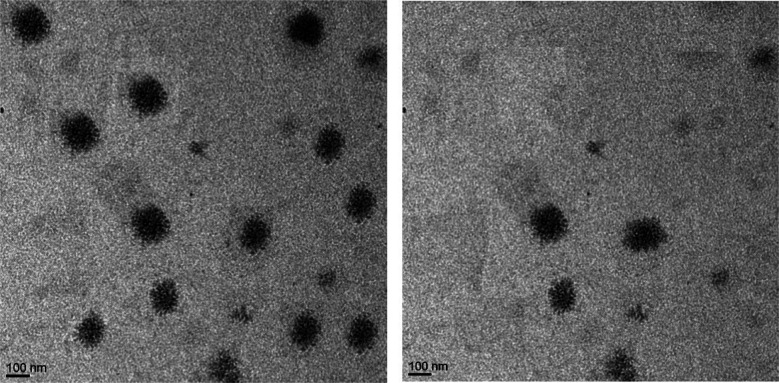
TEM images depict the shapes and size of liposomal emulgels containing *Eucalyptus* and clove EOs. Reprinted from ref. 190. Copyright MDPI 2023.

The outstanding anticancer properties of many EOs have encouraged other researchers to reveal the potential use of a combination of EOs co-encapsulated with chemotherapeutics. Hence, Pachauri *et al.* (2023) could successfully encapsulate the EOs of clove and *Eucalyptus* alongside the 5FU chemotherapeutic agent into liposomal emulgel nanocarriers. The obtained liposomal nanocarriers were revealed to have a spherical and smooth appearance within an approximate size of 100 nm, as determined by SEM and TEM images (Fig. 26).^190^ Clove EOs, belonging to *Syzygium aromaticum* of the Myrtaceae family, are commonly utilized in the food industry (*e.g.*, as a preservative) and in traditional medicine, mainly owing to eugenol, the major component of clove EOs, which has well-established anticancer, antimicrobial, antioxidant, and anti-inflammatory activities.^191,192^ Also, eugenol can be administered for patients subjected to chemotherapy as an adjunct therapy.^191^ Additionally, the EOs of *Eucalyptus* have demonstrated a wide variety of therapeutic properties including suppression of various tumor cells.^193^ Hence, the cytotoxic activity of the developed liposomal emulgel nanocarriers encapsulating the EOs alongside 5FU was investigated against skin cancer, B16-F10 mouse skin melanoma cells.^190^ The nanocarriers encapsulating clove EOs (5% w/w%), *Eucalyptus* EOs (5%), and 5FU (5%), utilizing a concentration of 1 mg mL^−1^ of the assessed formulations, could effectively target B16-F10 cells *via* enhancing the permeability and therapeutic efficacies of their cargos.^190^ Significantly, the liposomal nanocarriers containing the EOs of *Eucalyptus* and 5FU reduced the B16-F10 cell viability to 15.74 ± 11.78% compared to 61.53 ± 1.8% viable cells shown with cells treated with nanocarriers containing 5FU only and around 85% viable cells shown with cells treated with nanocarriers containing *Eucalyptus* EOs only.^190^ Similarly, the liposomal nanocarriers containing the EOs of clove and 5FU reduced the B16-F10 cell viability to 40.15 ± 15.54% compared to 61.53 ± 1.8% viable cells shown with cells treated with nanocarriers containing 5FU only and around 87% viable cells shown with cells treated with nanocarriers containing clove EOs only.^190^ Conversely, the liposomal formulations containing concentrations of 1%, 2%, and 3% of either the EOs and/or 5FU failed to show a significant reduction in the viability of the treated cells.^190^ These findings encourage the potential use of similar EOs coupled with other chemotherapeutic agents to reduce the required dose of the chemotherapy and hence its severe side effects.

**Fig. 27:**
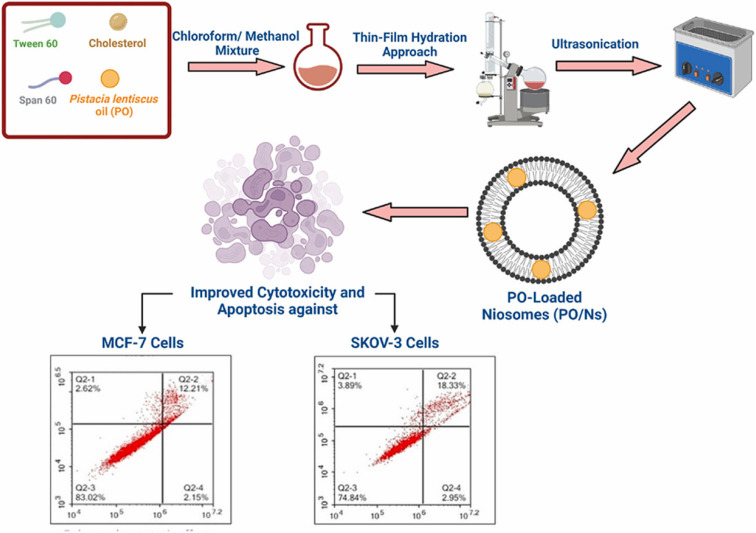
Schematic overview of the preparation of niosomes encapsulating *P. lentiscus* EOs, showing enhanced cytotoxicity against Skov-3 and MCF-7 cancer cells compared to free EOs. Reprinted from ref. 24. Copyright Elsevier 2023.

Fahmy *et al.* (2023) reported the successful encapsulation of *Pistacia lentiscus* EOs into niosomes, while investigating their cytotoxic activity against breast cancer MCF-7 and ovarian cancer Skov-3 cells, as shown in Fig. 27.^24^ GC-MS analysis depicted α-pinene (81.20%) as the predominant component of the obtained EOs. The resulting niosomes appeared in spherical shapes with a diameter of 120 nm, EE% of 93.4 ± 15.1%, *Z*-potential of −13.09 ± 2.9 mV, and PDI of 0.13 ± 0.06, reflecting a well-stable and monodispersed nanosystem with an excellent encapsulation efficiency.^24^ More importantly, the niosomes exhibited a 10-fold increase in the cytotoxicity of the encapsulated *P. lentiscus* EOs against Skov-3 and MCF-7 cancer cells, showing IC50 values of 4.88 μg mL^−1^ and 7.39 μg mL^−1^, respectively, compared to free EOs which exhibited an IC50 of 57.04 μg mL^−1^ against Skov-3 cells and an IC50 of 69.1 μg mL^−1^ against MCF-7 cells, after 72 h of incubation.^24^ Moreover, the niosomes depicted a substantial safety profile of the encapsulated EOs, showing insignificant cytotoxic activity against the normal breast MCF10A epithelial cells (IC50 > 200 μg mL^−1^). Furthermore, the flow cytometric analysis of niosomes encapsulating the EOs demonstrated a significant increase in the apoptotic effects in the Skov-3 and MCF-7 cells by 9-fold and 4-fold (combined apoptosis), respectively, compared to free EOs, after 48 h of treatment. Also, the niosomes boosted the necrotic activity of the encapsulated EOs against cancer cells by 8-fold compared to free EOs.^24^ Additionally, significant increases in sub-G1 cell populations were observed in cells treated with the encapsulated niosomes (12.42 ± 2.29 in Skov-3; 13.69 ± 1.01 in MCF-7 cells) compared to cells treated with free EOs (2.19 ± 0.15 in Skov-3; 1.42 ± 1.42 in MCF-7 cells), suggesting that the encapsulated niosomes exerted their prime cytotoxic effect by trapping the cells in the sub-G1 phase. Eventually, real-time PCR analysis further confirmed these findings, showing upregulation of the proapoptotic markers (Bak and Bax) and downregulation of the antiapoptotic marker (Bcl-2) in both Skov-3 and MCF-7 cells treated with EOs loaded into niosomes compared to cells treated with free EOs.^24^ These results emphasize the potential of niosomes as promising nanocarriers for the delivery of similar biomolecules against various tumor tissues.

**Fig. 28:**
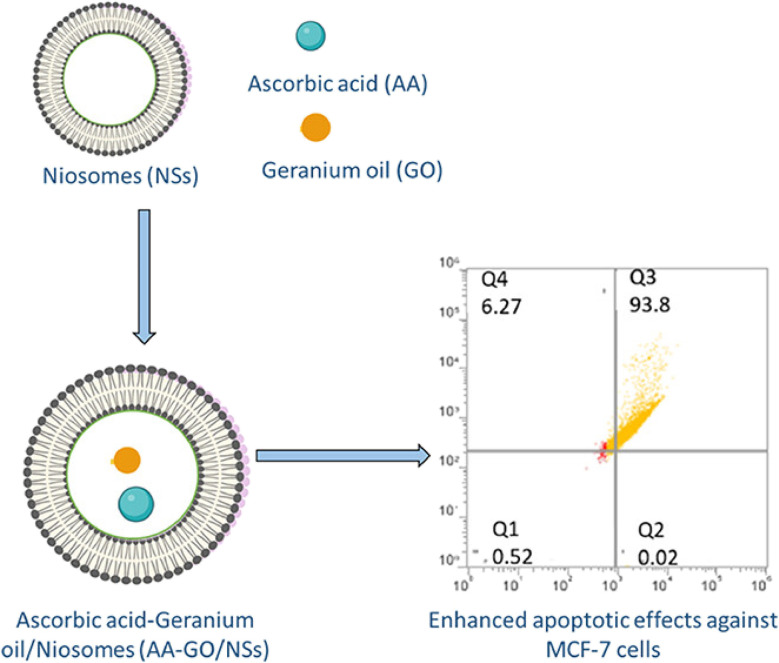
Schematic diagram illustrating niosomes co-encapsulating geranium EOs and ascorbic acid, showing enhanced apoptotic activity against MCF-7 cells in comparison to free EOs. Reprinted from ref. 194. Copyright ACS Omega 2023.

Fahmy *et al.* (2023) integrated geranium EOs and ascorbic acid into niosomes to examine their individual and combined synergistic effects on MCF-7 cells, as illustrated in Fig. 28. Dynamic light scattering and EE% of the prepared niosomes revealed that the combined niosomal nanoformulation encapsulating geranium EOs and ascorbic acid exhibited a diameter of 219.4 ± 44.5 nm, PDI of 0.21 ± 0.17, *Z*-potential of −6.39 ± 1.96 mV, and EE% of 98.3 ± 4.2% for EOs and 98.7 ± 3.1% for ascorbic acid.^194^ On the other hand, the geranium EO loaded niosomes had a diameter of 210.3 ± 35.0 nm, PDI of 0.24 ± 0.16, *Z*-potential of −9.81 ± 1.12 mV, and EE% of 98.1 ± 5.1%, and the ascorbic acid niosomes showed a diameter of 204.5 ± 29.8 nm, PDI of 0.19 ± 0.13, *Z*-potential of–7.45 ± 1.23 mV, and EE% of 99.5 ± 2.3%, indicating uniform and monodispersed nanosystems with excellent entrapment efficiency.^194^ For cytotoxicity analysis, MCF-7 cells were treated with the obtained niosomes for 24 h, in which the combination niosomes showed the highest cytotoxic effect (IC50 of 7.69 ± 8 μg mL^−1^), followed by ascorbic acid niosomes (IC50 of 18.97 ± 6 μg mL^−1^), compared to free ascorbic acid (IC50 of 20.5 ± 13 μg mL^−1^), and then geranium EO niosomes (IC50 of 47.46 ± 11 μg mL^−1^), compared to free EOs (IC50 of 65.63 ± 14), indicating enhanced cytotoxic effects upon encapsulation into niosomes, with synergistic anticancer activity observed with the combination.^194^ Additionally, the apoptotic effects on MCF-7 cells were investigated using flow cytometry in which ascorbic acid niosomes induced predominantly late apoptosis (viable: 3.85%, early apoptotic: 0%, and late apoptotic: 79.1%) and geranium EO niosomes resulted in the following percentages of viability (viable: 2.12%, early apoptotic: 0%, and late apoptotic: 87.3%). However, combined niosomes induced significant levels of apoptosis, with a decrease in viable cells to 0.52%, and increase in late apoptotic cells to 93.8%, compared to the control (viable: 98.8%).^194^ Furthermore, ROS production was assessed using the fluorogenic dye H2DCFDA. All the prepared niosomes led to a significant decrease in fluorescence, indicating reduced ROS production. However, the combination niosomes showed the most significant decrease in fluorescence compared to other treatments, suggesting the highest antioxidative activity.^194^ These findings suggest the potential of combined niosomal formulations, including EOs, as promising therapeutic agents against breast cancer.

### 5.2. Polymeric nanoparticulate systems

**Fig. 29:**
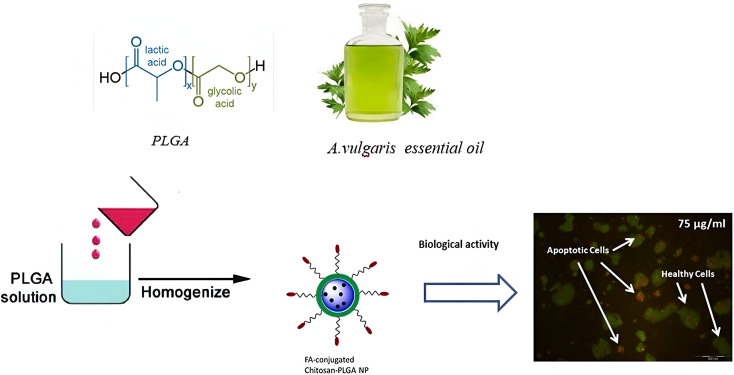
Schematic diagram showing the apoptotic activity of modified PLGA nanoparticles encapsulating *A. vulgaris* EOs against HT-29 colon cancer cells. Reprinted with permission from ref. 195. Copyright Elsevier 2022.

Alirezaei *et al.* (2022) formed PLGA nanoparticles encapsulating the EOs of *Artemisia vulgaris* to assess their anticancer activity against colon adenocarcinoma cells (HT-29), as illustrated in Fig. 29.^195^ The surfaces of PLGA nanoparticles were modified with chitosan and folic acid conjugates to enhance targeting efficiency. Eventually, the obtained nanoparticles had a particle size of 298.96 nm, PDI of 0.055, *Z*-potential of +20 mV, and EE% of 99.79%, indicating a stable and homogeneous nanoparticle system accompanied by remarkable encapsulation efficiency.^195^ The nanoparticles showed significant cytotoxic effects of the encapsulated *A. vulgaris* EOs against HT-29 cancer cells with inhibition rates of 10%, 20%, and 80% of cells treated with concentrations of 25 μg mL^−1^, 50 μg mL^−1^, and 100 μg mL^−1^ of encapsulated EOs, respectively. Conversely, the encapsulated nanoparticles maintained 100% cell viability of HFF normal cells (normal fibroblasts), indicating a superior therapeutic profile of the developed nanoparticles accompanied by superior selectivity towards malignant cells. Moreover, the encapsulated nanoparticles showed an apoptotic activity of 59% against HT-29 cells using a concentration of 75 μg mL^−1^ of EOs.^195^ Furthermore, the encapsulated nanoparticles could depict a notable antiangiogenetic activity reducing both the length, from 35 mm to ∼23 mm, and number, from 30 to 20 vessels of the treated blood vessels with 2 mg mL^−1^ of encapsulated EOs.^195^ These results could significantly encourage the use of similar nanosystems for encapsulation of other EOs with promising therapeutic efficacies.

**Fig. 30:**
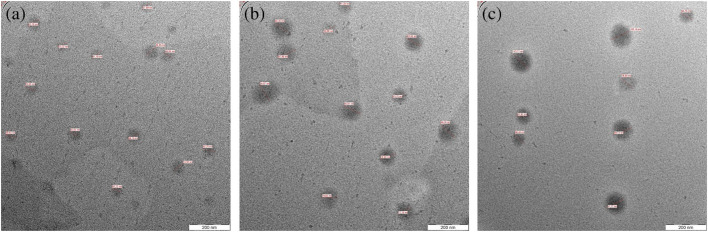
TEM images of chitosan nanoparticles encapsulating EOs of *C. cauliflora* extracted from the (a) twig, (b) leaf, and (c) fruit. Reprinted with permission from ref. 196. Copyright Elsevier 2022.

Chitosan is a natural biopolymer, derived from chitin *via* deacetylation, with promising biodegradability and compatibility profiles and minimal toxicity. Chitosan is FDA-approved (2001) and recognized as a GRAS material.^196^ Hence, Samling *et al.* (2022) developed three chitosan nanoparticulate systems to encapsulate the EOs extracted from the twigs, leaves, and fruits of *Cynometra cauliflora*, aiming to augment their cytotoxic properties against breast cancer.^196^ The obtained chitosan nanoparticulate systems encapsulating *C. cauliflora* EOs were revealed to have a well-dispersed and spherical appearance with a diameter range of 10.02 to 14.68 nm and a PDI range of 0.245 to 0.628, as presented in the TEM images in Fig. 30. Additionally, the EE% fell between 38.83% and 44.16%, and the nanoparticles carried negative surface charges (−17.6 to −21.1 mV).^196^ More importantly, cytotoxicity assessments were conducted on MCF-7 and MDA-MB-231 breast cancer cells. For MCF-7 cells, chitosan nanoparticles loaded with EOs derived from the leaves and twigs demonstrated significant cytotoxicity, with IC50 values of 3.72 μg mL^−1^ and 7.69 μg mL^−1^, respectively, following 72 h of treatment. This efficacy was further supported by the significant elevation (>10) in the selective cytotoxicity index (SI) with values of 26.88 (leaves) and 13 (twigs) against MCF-7 cells, respectively, after 72 h incubation.^196^ Conversely, chitosan nanoparticles loaded with the EOs of *C. cauliflora* fruits exhibited lower cytotoxic activity, displaying an IC50 value of 17.81 μg mL^−1^ and SI of 5.61 against MCF-7 cells. Moreover, for MDA-MB-231 cells, all chitosan nanoparticles loaded with either of the EOs exhibited moderate cytotoxicity, with IC50 values ranging from 16.24 to 17.65 μg mL^−1^ after 72 hours of treatment. Notably, no cytotoxic effects were observed against the normal cell line MCF-10A.^196^ It can be concluded that the encapsulation of EOs into chitosan nanoparticles substantially improved their cytotoxic activities while securing superior stability and biocompatibility profiles.

**Fig. 31:**
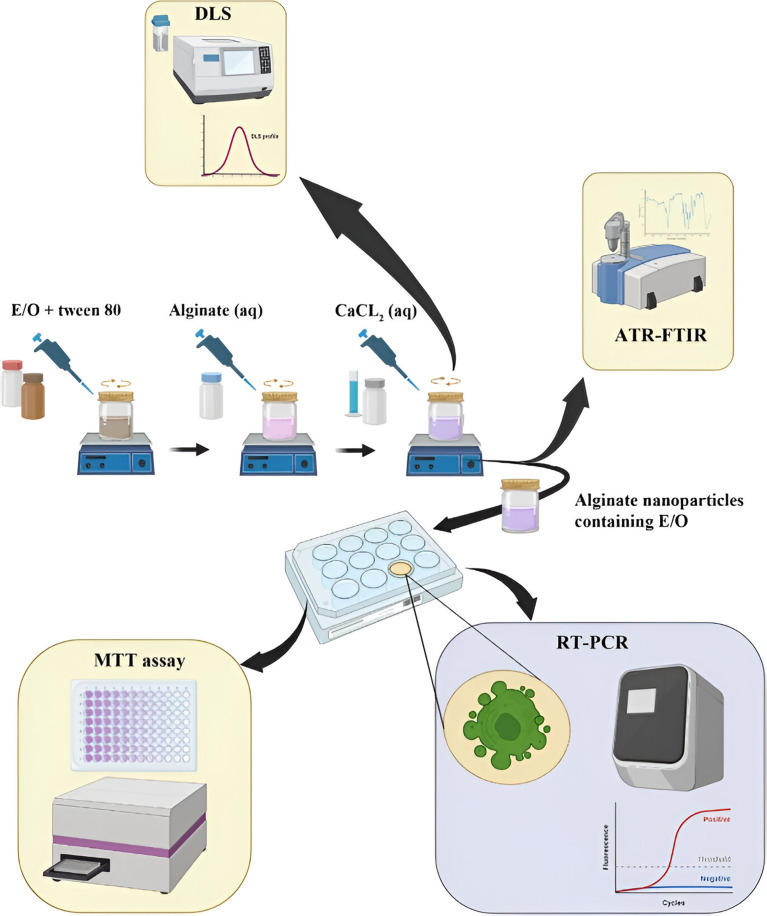
Schematic illustration depicts the development of two alginate nanoparticulate systems loaded with clove EOs (*Syzygium aromaticum*) and eugenol alongside their characterization tests conducted and anticancer activity investigated against breast cancer MCF-7 and melanoma A-375 cells. Reprinted with permission from ref. 197. Copyright Springer Nature 2023.

Alginate is a natural biopolymer, a linear polysaccharide derived from alginic acid, and has potential as a nanocarrier due to its established biodegradable and biocompatible properties.^197^ Yarian *et al.* (2023) developed two alginate nanoparticulate systems loaded with clove EOs (*Syzygium aromaticum*) (Alg-clove-NPs) and eugenol (Alg-eug-NPs) and examined their anticancer properties against breast cancer MCF-7 and melanoma A-375 cells (Fig. 31).^197^ Employing a GC-MS analysis of the obtained EOs of clove, eugenol (66%) was detected as the major compound. Interestingly, both nanosystems showed stable profiles with Alg-clove-NPs depicting a particle size of 122 ± 7 nm and PDI of 0.33, whereas Alg-eug-NPs exhibited a particle size of 87 ± 8 nm and PDI of 0.25.^197^ More significantly, both nanosystems exhibited remarkable anticancer activities against MCF-7 and A-375 cells, with Alg-clove-NPs showing IC50 values of 321.9 μg mL^−1^ and 358.3 μg mL^−1^, respectively, after 24 h of incubation with cells. Alg-eug-NPs,^197^ on the other hand, depicted IC50 values of 328.8 μg mL^−1^ and 757.5 μg mL^−1^ against MCF-7 and A-375 cells, respectively, after 24 h of incubation. Additionally, the investigation into the gene expression patterns of Bax and Bcl-2 in MCF-7 and A-375 cells after their exposure to both nanosystems unveiled an elevation in their ratio of Bax/Bcl-2 (>1), indicating a significant increase in apoptosis in the treated cancer cells.^197^ Current findings highlight the potential of polymeric alginate nanoparticles as nanocarriers for application against breast and melanoma cancers.

**Fig. 32:**
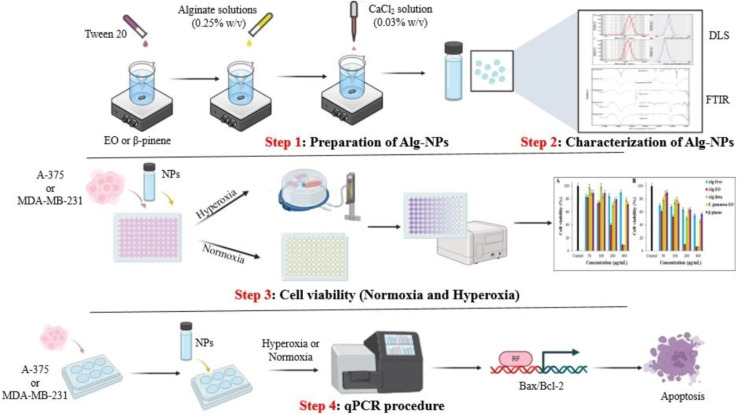
Schematic diagram illustrating alginate-based nanoparticles encapsulating β-pinene and *Ferula gummosa* EOs, demonstrating enhanced cytotoxicity against A-375 and MDA-MB-231 cells under normoxic and hyperoxic conditions, with significant apoptotic activity evidenced by increased Bax/Bcl-2 ratios. Reprinted from ref .^198^. Copyright Springer Nature 2023.

Similarly, Osanloo *et al.* (2023) successfully developed two alginate-based polymeric nanoparticles encapsulating β-pinene and the EOs of *Ferula gummosa*, aiming to investigate their potential in combating human melanoma (A-375) and breast cancers (MDA-MB-231), under the conditions of both normal oxygen levels (normoxia) and at elevated oxygen levels (normobaric hyperoxia), as illustrated in Fig. 32.^198^ GC-MS identified β-pinene (61.57%) as the predominant compound in the obtained *F. gummosa* EOs. The obtained nanosystems of alginate nanoparticles encapsulating β-pinene (Alg-β-pinene-NPs) and *F. gummosa* EOs (Alg-EOs-NPs) showed particles with sizes of 174 ± 7 and 137 ± 6 nm, respectively, accompanied by *Z*-potential values of 12.4 ± 0.7 and 28.1 ± 1 mV, respectively, reflecting well-stable profiles of the obtained nanoparticles.^198^ More importantly, the cytotoxic assessments conducted on MDA-MB-231 cells treated with free EOs and Alg-EOs-NPs, under normoxic conditions, showed IC50 values of 184 μg mL^−1^ and 104 μg mL^−1^, respectively. For the MDA-MB-231 cells treated with free β-pinene and Alg-β-pinene-NPs under normoxic conditions, the IC50 values were 323 μg mL^−1^ and 142 μg mL^−1^, respectively, after 24 h of incubation.^198^ On the other hand, under hyperoxic conditions, free EOs and Alg-EOs-NPs showed IC50 values of 194 μg mL^−1^ and 120 μg mL^−1^, respectively, against MDA-MB-231 cells. β-Pinene and Alg-β-pinene-NPs showed IC50 values of 536 μg mL^−1^ and 205 μg mL^−1^, respectively, against MDA-MB-231 cells, after 24 h of incubation. These findings refer to the significant increase in the cytotoxic activities of both *F. gummosa* EOs and β-pinene upon their encapsulation into alginate nanoparticles, with the higher stability, biocompatibility, and sustainability profiles attributed to the alginate polymeric nanoparticles.^198^ Furthermore, the cytotoxic activities of free *F. gummosa* EOs and Alg-EOs-NPs, under normoxic conditions, showed IC50 values of 262 μg mL^−1^ and 136 μg mL^−1^, respectively, against A-375 cells. For the A-375 cells treated with free β-pinene and Alg-β-pinene-NPs under normoxic conditions, the IC50 values were 302 μg mL^−1^ and 248 μg mL^−1^, respectively, after 24 h of incubation. On the other hand, under hyperoxic conditions, free EOs and Alg-EOs-NPs showed IC50 values of 226 μg mL^−1^ and 76 μg mL^−1^, respectively, against A-375 cells. β-Pinene and Alg-β-pinene-NPs showed IC50 values of 242 μg mL^−1^ and 132 μg mL^−1^, respectively, against A-375 cells, after 24 h of incubation.^198^ These results indicate a substantial increase in the cytotoxic activities of both *F. gummosa* EOs and β-pinene upon their encapsulation into alginate nanoparticles against melanoma, with the higher stability, biocompatibility, and sustainability profiles attributed to the alginate polymeric nanoparticles. Also, hyperoxic conditions might further enhance the cytotoxic activities of the tested compounds, attributed to the increased oxidative stress and apoptosis induction that accompany such conditions helping prevent the proliferation and survival of cancer cells.^198^ Moreover, Alg-EOs-NPs and Alg-β-pinene-NPs showed significant induction in the apoptosis in MDA-MB-231 and A-375 cells confirmed by an increased Bax/Bcl-2 ratio, as evidenced by the upregulation of Bax (a pro-apoptotic protein) and the downregulation of Bcl-2 (an anti-apoptotic protein).^198^

**Fig. 33:**
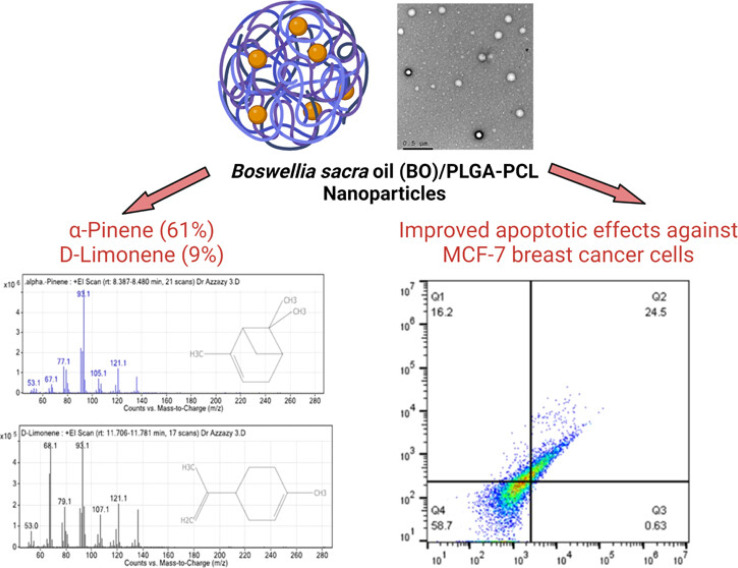
Schematic diagram illustrating PLGA-PCL nanoparticles encapsulating *B. sacra* EOs, highlighting major compounds detected (α-pinene and d-limonene), and their enhanced cytotoxicity and apoptotic activity against MCF-7, with increased necrosis and significant cell cycle arrest at the G0-G1 phase. Reprinted from ref. 199. Copyright ACS Omega 2022.

Azzazy *et al.* (2023) effectively encapsulated the EOs of *Boswellia sacra* into PLGA-PCL nanoparticles to combat breast cancer (Fig. 33).^199^ The resins of *B. sacra* were initially utilized to extract their corresponding EOs *via* a green extraction method of hydrodistillation and they were then encapsulated into PLGA-PCL.^199^ The chemical composition of the extracted EOs was analyzed using GC-MS, exhibiting a major component of α-pinene (61.05%) followed by d-limonene (9%).^199^ Notably, α-pinene has been well-documented for its anticancer properties against various malignancies.^20,200,201^ The obtained nanoparticles showed spherical shapes with a size of 230.3 ± 3.7 nm, *Z*-potential of −20.36 ± 4.89 mV, EE% of 80.59 ± 3.37%, and PDI of 0.13 ± 0.03, indicating a uniform particle size distribution and favorable stability with an excellent encapsulation efficiency of the obtained nanosystem.^199^ More importantly, the encapsulated nanoparticles depicted higher cytotoxic activity against breast cancer cells (MCF-7) with an IC50 of 2.32 ± 0.49 μg mL^−1^ compared to free EOs which showed an IC50 of 9.55 ± 0.7 μg mL^−1^.^199^ Furthermore, the encapsulated nanoparticles demonstrated a significant increase in the apoptotic activity of the EOs against MCF-7 cells (24.5%) compared to free EOs (12.7%).^199^ Also, the encapsulated nanoparticles exhibited a more than two-fold increase in the necrotic cells (16.2%) compared to free EOs (7.4%).^199^ Moreover, the encapsulated nanoparticles exhibited an 84% cell cycle arrest of MCF-7 cells, at the G0–G1 phase, compared to 62.6% shown with MCF-7 cells treated with free EOs.^199^ These findings indicate the great anticancer potential of the developed nanoparticles not only in inducing cancer cell death but also in interfering with their progression.

Rajivgandhi *et al.* (2023) developed chitosan nanoparticles encapsulating *Aegle marmelos* EOs and evaluated their anticancer activity against lung cancer.^202^ The formation of the nanoparticles and encapsulation of the EOs were confirmed *via* SEM and TEM examinations. For the SEM analysis, images showed the encapsulated nanoparticles with homogeneous, round-shaped, and rough surfaces, compared to free chitosan which displayed a rock-shaped structure. For the TEM analysis, images of the encapsulated nanoparticles depicted spherical nanovesicles (<100 nm), with intrinsic tendency for agglomeration, and thin layers surrounding the obtained nanovesicles, referring to the successful encapsulation of the *A. marmelos* EOs.^202^ More importantly, the encapsulated nanoparticles could effectively improve the cytotoxicity of the EOs against lung cancer cells (A549) by two-fold compared to free EOs of *A. marmelos*.^202^ Also, using a phase contrast microscope, the encapsulated nanoparticles could induce a significant apoptotic activity against the treated cancer cells which might explain the enhanced cytotoxic activity revealed above.^202^ These findings suggest the potential use of chitosan nanoparticles for encapsulation of EOs, owing to the intrinsic therapeutic activity accompanied by well-established biocompatibility of the chitosan nanoparticles.

**Fig. 34:**
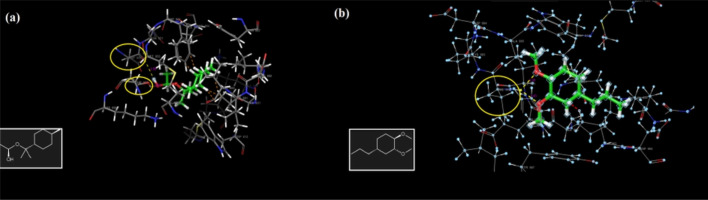
Molecular interactions showing (a) methyleugenol binding to valine at position 882 and (b) α-terpinyl acetate binding to aspartic acid at position 950 *via* hydrogen bonds within the PI3K/mTOR target receptor. Reproduced from ref. 203. Copyright MDPI 2022.

Ercin *et al.* (2022) reported the synthesis of PLGA nanoparticles loaded with the EOs of *Laurus nobilis* and investigated their anticancer activity.^203^ The obtained PLGA nanoparticles loaded with the EOs of *L. nobilis* exhibited an average particle size of 211.4 ± 4.031 nm, *Z*-potential of −7.87 ± 1.15 mV, and PDI of 0.068 ± 0.016, reflecting a well-stable and monodispersed suspension of the obtained particles.^203^ The EE% was calculated to be 59.25%, while the loading capacity was determined to be 25.65%. To investigate the anticancer activity of the prepared PLGA nanoparticles, a DNA binding assay using UV-VIS titration was employed to assess the interaction between the encapsulated nanoparticles and DNA.^203^ The analysis revealed a 6 nm bathochromic shift, indicating a shift towards longer wavelengths, along with a significant 93.80% reduction in absorbance, suggesting a hypochromic effect. These findings suggest notable alterations in the electronic structure of DNA, highlighting the potential therapeutic efficacy of the encapsulated nanoparticles in cancer treatment.^203^ Moreover, molecular docking analyses demonstrated interactions between the encapsulated nanoparticles and the dual inhibitor PI3K/mTOR, crucial regulators of cancer cell growth and proliferation. Particularly, two components of *L. nobilis* EOs which are α-terpinyl acetate and methyleugenol bonded to aspartic acid at position 950 and valine at position 882, respectively, through hydrogen bonds within the PI3K/mTOR target receptor, as shown in Fig. 34.^203^ These observations support the potential of these nanoparticles as therapeutic agents for cancer treatment, providing valuable insights into their mechanism of action at the molecular level.

### 5.3. Nanoemulsions

Edris and Abd-Rabou (2022) developed two distinct oil-in-water (O/W) nanoemulsions containing *B. sacra* EOs and investigated their anticancer activities against lung cancer A549 cells.^204^*B. sacra* EOs, obtained *via* hydrodistillation, predominantly comprised α-pinene (59.5 ± 0.9)%, as identified by GC-MS. Consequently, the EOs were encapsulated into two separate nanoemulsions.^204^ Both nanoemulsions were composed of *B. sacra* EOs (5%) and two surfactants of Cremophor RH40 and sunflower oil, whereas the second nanoemulsion system included an additional surfactant propylene glycol (1%). Both nanoemulsions exhibited similar PDI values of 0.3, whereas they differed in diameter, with the propylene glycol-containing nanoemulsion measuring 28.7 ± 1.9 nm and the propylene glycol-free nanoemulsion measuring 95.57 ± 0.3 nm.^204^ Interestingly, the EOs formulated in both nanoemulsions exhibited substantially higher cytotoxic activities against A549 cells with IC50 values of 22.22 μg mL^−1^ (for the propylene glycol-free nanoemulsion) and 14.88 μg mL^−1^ (for the propylene glycol-containing nanoemulsion), compared to free EOs which showed an IC50 of 34.60 μg mL^−1^, following 72 h of incubation.^204^ Remarkably, the addition of propylene glycol might have significantly augmented the cytotoxic efficacy of the obtained nanoemulsion, likely by enhancing membrane permeability and fluidity of the nanoparticles to eventually improve the targeting ability of the encapsulated EOs.^204^ Flow cytometric analysis showed that both nanoemulsions could effectively upregulate several pro-apoptotic genes (Bax, P53, Caspase 8, FAAD, and DR5) and downregulate several anti-apoptotic genes (STAT-3, NF-kB, and Bcl-2) in treated cancer cells.^204^ Furthermore, both nanoemulsions could markedly increase the levels of reactive oxygen species produced in treated cells, represented by nitric oxide (NO) and the inducible nitric oxide synthase (iNOS) enzyme. The propylene glycol-containing nanoemulsion showed an increase in the NO and iNOS in the treated cells to reach a level of 46.54 and 27.64, respectively, compared to the untreated control with 15.6 and 6.7, respectively. Also, the propylene glycol-free nanoemulsion showed an increase, to a lower extent, in the levels of NO and iNOs in the treated cells with 31.37 and 18.39, respectively.^204^ These results indicate the superior anticancer activities of the encapsulated *B. sacra* EOs imparted by the proposed nanoemulsions which could further be augmented by *in vivo* studies.

**Fig. 35:**
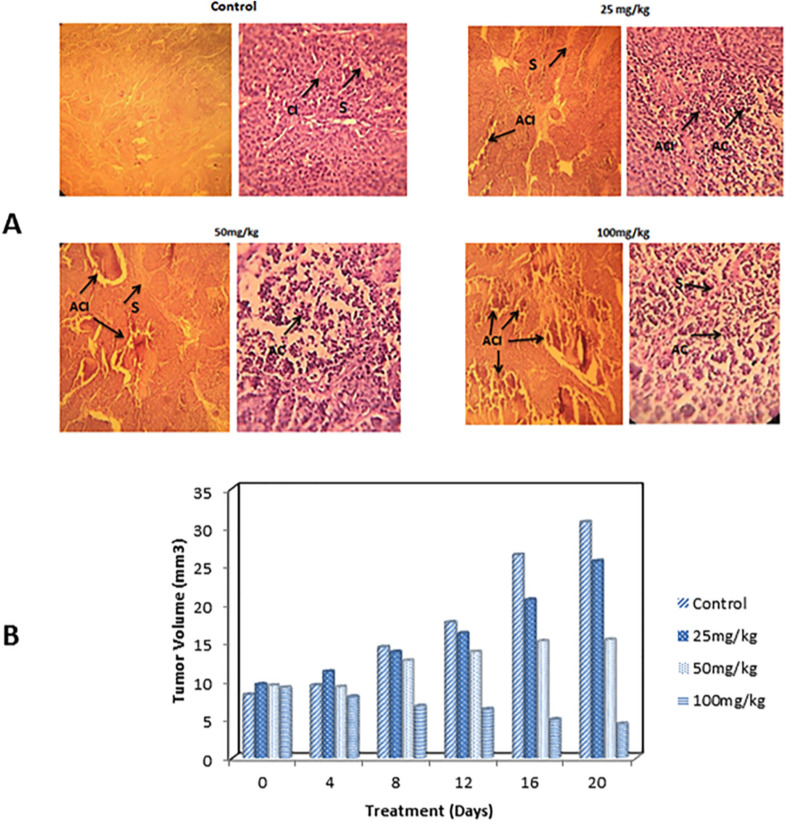
This figure shows the effects of different concentrations of the nanoemulsion encapsulated with *Ferula assa-foetida* EOs on breast tumors in murine mice after 20 days. (A) Changes in the tumor tissue structure, with treated groups displaying reduced tumor cell clusters and increased cell death compared to the untreated control. (B) Tumor size measurements over 20 days of treatment using different doses of the encapsulated nanoemulsion. Abbreviations: CI = carcinoma islands, S = stromal, ACI = apoptotic carcinoma islands, and AC = apoptotic cells.^205^ Reprinted with permission from ref. 205. Copyright Taylor & Francis 2022.

Azani *et al.* (2022) developed an O/W nanoemulsion incorporating *Ferula assa-foetida* EOs to assess their anticancer properties against both melanoma (A-2058) and breast cancer cells (MCF-7).^205^ The obtained nanoemulsion droplets exhibited an average diameter of 26.51 nm, PDI of 0.257, and *Z*-potential of −38.26 mV, indicating a uniform size distribution of the nanoemulsion with excellent stability.^205^ The obtained nanoemulsions showed remarkable cytotoxic activity against breast cancer (MCF-7) and skin cancer cells (A-2058) with IC50 values of 64.42 μg mL^−1^ and 201.85 μg mL^−1^, respectively, following 48 h incubation. Also, the nanoemulsions depicted an excellent safety margin profile against the normal cells of HFF (IC50 > 350 μg mL^−1^) and HUVEC (IC50 > 400 μg mL^−1^).^205^ The nanoemulsions could also induce substantial apoptosis of MCF-7 cells, as revealed by the significant upregulation of Bax gene expression, at a concentration of 125 μg mL^−1^, and downregulation of Bcl-2 gene expression using a concentration of 32 μg mL^−1^.^205^ Moreover, the nanoemulsions exhibited significant anti-angiogenic effects shown *via* the suppression of VEGF and VEGFR gene expression among MCF-7 cells treated with 32 μg mL^−1^ of encapsulated EOs.^205^ The nanoemulsion showed a significant apoptotic activity against the murine breast cancer model. After 20 days of the *in vivo* assessments, the tumor volume in the control group reached 32 mm^3^, while it reduced remarkably in the treated mice reaching 26 mm^3^, 16 mm^3^, and 4 mm^3^ with the corresponding administered doses of the encapsulated EOs of 25 mg kg^−1^, 50 mg kg^−1^, and 100 mg kg^−1^ (Fig. 35).^205^ These results encourage the potential use of similar nanosystems for targeting other tumor tissues.

**Fig. 36:**
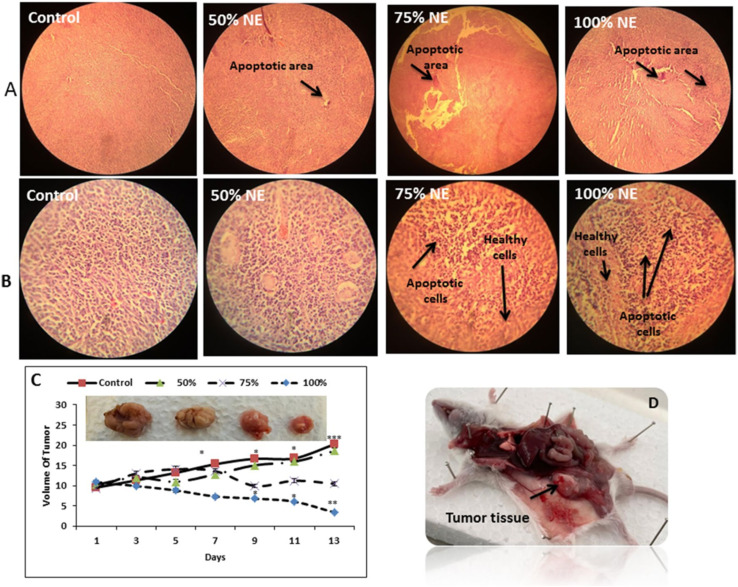
Therapeutic effects of nanoemulsions loaded with the EOs of *Ferula gummosa* (NE) on tumor growth in a mouse model of colon cancer. (A & B) Histopathological images of tumor tissues treated with the NE compared to the control group. (C) Changes in the volume of the tumor samples treated with NE as compared to the control sample. (D) The tumor tissue isolated from the mouse model. Reproduced with permission from ref. 206. Copyright Springer Nature 2022.

Nosrat *et al.* (2022) successfully formed a promising nanoemulsion system encapsulating the EOs of *Ferula gummosa* to assess their anti-tumor effects against colon cancer (HT-29).^206^ The obtained nanoemulsion exhibited a uniform droplet size of 24.6 nm, PDI of 0.41, and *Z*-potential of −28.5 mV, imparting the developed nanoemulsion with favorable stability and uniformity.^206^ The obtained nanoemulsion droplets encapsulating the EOs exhibited remarkable cytotoxic activity against HT-29 cancer cells with an IC50 value of 1.087 μg mL^−1^, while showing insignificant inhibitory effects against normal HFF cells, following 72 h of treatment.^206^ Furthermore, the nanoemulsion encapsulating the EOs induced remarkable apoptosis and angiogenesis reduction in treated HT-29 cells. This was evidenced by the significant upregulation of the gene expression of CAT and SOD by 4.48- and 2.5- fold, respectively, inducing higher antioxidant activities. Also, the gene expression levels of the apoptotic markers of Caspase-3, Bax, and Caspase-9 increased by 1.89-, 2.58-, and 4.37- fold, respectively.^206^ Additionally, significant downregulation was reported with the genes of the anti-apoptotic Bcl-2 marker by 0.19-fold and the angiogenesis VEGF marker by 0.16-fold. Moreover, nanoemulsions encapsulating EOs were administered to murine colon cancer mice models, showing significant reduction in the tumor volume by 69.72% after 14 days of treatment (Fig. 36).^206^ These findings indicate the promising anticancer effects of *F. gummosa* EO loaded nanoemulsions and encourage their further use against other sorts of cancers.

Abadi *et al.* (2022) prepared a nanoemulsion system containing the EOs of clove (*Syzygium aromaticum* L.) and evaluated their anti-tumor activity against colon cancer cells (HT-29).^207^ The GC-MS analysis of the extracted EOs exhibited eugenol as the prime component (69.8%). The obtained nanoemulsion had a size of 131.2 nm, PDI of 0.248, and *Z*-potential of −42.1 mV, indicating a stable and monodispersed nanosystem.^207^ On the other hand, the cytotoxic analysis of nanoemulsion showed an IC50 of 74.8 μg mL^−1^ against HT-29, after 48 h incubation. Moreover, the nanoemulsion showed a promising apoptotic activity in HT-29 cells with a 5-fold increase in the gene expression of the apoptotic response effector, Caspase-3, following 10 h of incubation.^207^ More importantly, the *in vivo* study reported that administration of 10 mg kg^−1^ and 20 mg kg^−1^of nanoemulsions encapsulating EOs showed a remarkable safety profile, exhibiting insignificant effects in the histopathological analysis of the intestine, liver, and kidney in the treated mice. Also, the nanoemulsion boosted the gene expression of the antioxidant enzymes of SOD, CAT, and GPx by 3.5-, 2.4-, and 1.8- fold, respectively, in the liver tissues of the treated mice, reducing harmful free radicals and protecting healthy cells.^207^

**Fig. 37:**
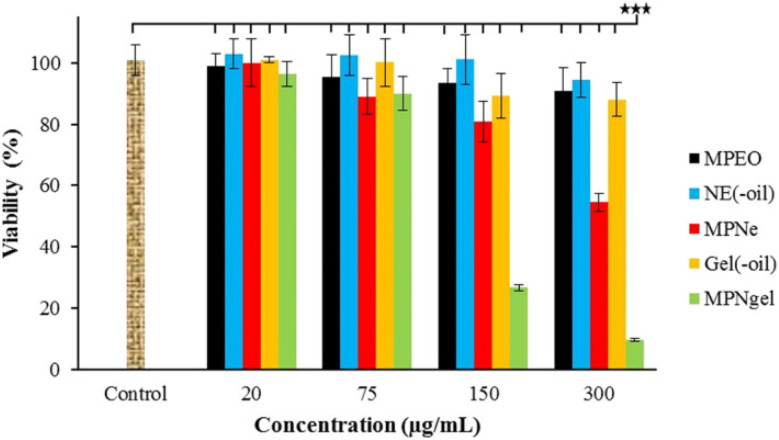
Cytotoxic properties of *Mentha pulegium* EOs (MPEO), free emulsion (NE(-oil)), free nanoemulsion (MPNe), blank nanogel (gel(-oil)), and nanogel (MPNgel) on A375 cells. Reprinted from ref. 208. Copyright Springer Nature 2023.

Azadi *et al.* (2023) developed two delivery nanosystems encapsulating *Mentha pulegium* EOs (MPEO) with promising anticancer activities shown against melanoma cells (A375).^208^ The first nanosystem encompassed a nanoemulsion designed to disperse the EO droplets (MPNe), whereas the second nanosystem was a nanogel (MPNgel) that formed a structured gel matrix for sustained EO release, utilizing carboxymethylcellulose (2% w/v) as a crosslinker. GC-MS analysis showed pulegone (68.11%) to be the major compound in the EOs. The nanoemulsion exhibited spherical droplets, with a size of 7.70 ± 1 nm, and a remarkable stability over a 9-month interval showing no signs of sedimentation or phase separation, at room temperature, securing a stable droplet size of 7.98 nm after a 9 months of storage.^208^ On the other hand, the nanogel system displayed favorable rheological behavior and robust stability, showing no signs of phase separation, sedimentation, or creaming of the nanogel while being stored at 4 °C and at room temperature over a 6-month period. More significantly, *M. pulegium* encapsulated within nanoemulsion and nanogel formulations, compared to their blank forms (NE oil and NE gel, respectively), demonstrated notable cytotoxicity at a concentration of 300 μg mL^−1^. This resulted in a 45% reduction in viability for nanoemulsion-loaded oils and a 90% reduction for nanogel-loaded oils against melanoma A375 cells, after 24 hours of incubation. This efficacy was significantly higher compared to their free EOs, as depicted in Fig. 37.^208^ Conversely, the free nanoemulsion, EOs, and nanogel failed to exhibit any significant cytotoxic activities against A375 cells. Moreover, flow cytometric analysis of the EOs encapsulated into nanogels induced early apoptosis of the treated A375 cells by 14% compared to 32% early apoptosis shown in cells treated with the EOs encapsulated into nanoemulsions, after 24 h of treatment.^208^ Eventually, gene expression analysis showed significant upregulation of the pro-apoptotic gene Bax and downregulation of the anti-apoptotic gene Bcl-2 in cells treated with both nanosystems, compared to the control and free *M. pulegium* EOs. These findings show the advantageous properties of EOs loaded into nanoemulsions and nanogels in enhancing the therapeutic efficacy and physicochemical properties of the loaded EOs.^208^ Overall, nanoemulsions are considered as promising drug delivery carriers for EOs, augmenting their cytotoxic properties while minimizing their drawbacks.

Karkanrood *et al.* (2022) synthesized a nanoemulsion encapsulating the EOs extracted from the fruits of *Pistacia atlantica*, aiming to explore its anticancer properties against PC3 (prostate cancer cells), A549 (lung cancer), and A2058 (melanoma cells).^209^ The obtained nano-sized emulsion system displayed dimensions of 35.8 nm, a PDI of 0.4, and a *Z*-potential of −32 mV, indicating a well-stable nanosystem.^209^ The cytotoxicity evaluation of the nanoemulsion encapsulated EOs displayed significant inhibitory effects against A2058, A549, and PC3 cells, after 48 h incubation, with IC50 values of 8.82 μg mL^−1^, 4.3 μg mL^−1^, and 2.87 μg mL^−1^, respectively.^209^ Furthermore, extended exposure of A549 cells to the obtained nanoemulsion demonstrated increased potency, with IC50 values of 7.7 μg mL^−1^, 4.3 μg mL^−1^, and 3.5 μg mL^−1^ at 24-, 48-, and 72 h post-treatment.^209^ Also, treatment with the obtained nanoemulsion notably increased intracellular ROS levels in A549 cells, reaching approximately 80% at a concentration of 10 μg mL^−1^, suggesting its capacity to induce DNA damage and apoptosis in cancer cells. Analysis of apoptotic gene expression in A549 cells revealed significant upregulation of Cas3, Cas-8, and IL-10 by 3.8-, 6.69-, and 7.25-fold, respectively, utilizing a concentration of 4.3 μg mL^−1^.^209^ Moreover, treatment of chick embryo chorioallantoic membranes with 100 μg mL^−1^ resulted in a significant reduction in blood vessel length (20.27 mm) compared to the control (38.4 mm). Similarly, the weight of treated embryos decreased to 2.57 g as compared to the control groups which showed a weight of 3.19 g, employing the same concentration.^209^ The developed *P. atlantica* EO loaded nanosystem has the potential for cancer treatment, and further *in vivo* assessments are encouraged to investigate their therapeutic efficacy.

### 5.4. Solid lipid nanoparticles

**Fig. 38:**
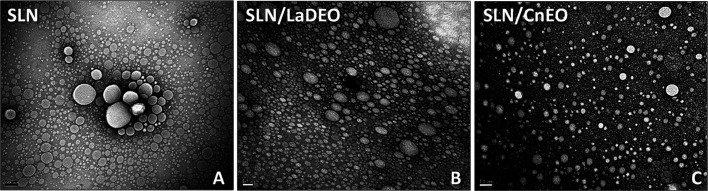
TEM images with a scale bar of 200 nm showing (A) solid lipid nanoparticles (SLN), (B) SLN containing *L. alba* EOs (SLN/LaDEO), and (C) SLN containing *C. nepeta* EOs (SLN/CnEO). Reprinted from ref. 210. Copyright MDPI 2023.

Rodenak-Kladniew (2023) *et al.* fabricated solid lipid nanoparticles (SLN) encapsulating EOs extracted from *Clinopodium nepeta* and *Lippia alba* to assess their cytotoxic efficacy against human lung cancer cells (A549) and colon cancer cells (HCT-116).^210^ GC-MS analysis showed pulegone (37.22%) to be the major component in *C. nepeta* EOs and dihydrocarveol isomer 1 (29.6%) in *L. alba* EOs.^210^ Individual encapsulation of each EO within SLN resulted in highly stable, monodispersed, and spherical nanoparticles. SLN containing *C. nepeta* EOs (SLN/CnEO) had a diameter of 141 nm, PDI of 0.2, and *Z*-potential of −5 mV, whereas SLN containing *L. alba* EOs (SLN/LaEO) exhibited a diameter of 151 nm, PDI of 0.2, and *Z*-potential of −6 mV.^210^ Additionally, Fig. 38 shows the TEM images of SLN, SLN/LaEO, and SLN/CnEO.^210^ The obtained SLN/CnEO could significantly augment the cytotoxic activity of the encapsulated EOs against A549 cancer cells with an IC50 of 66 ± 5 μL/L, compared to free *C. nepeta* EOs which exhibited an IC50 of 205 μL/L. Also, SLN/CnEO exhibited promising cytotoxic activity against HCT-116 cancer cells with an IC50 of 134 ± 11, compared to free *C. nepeta* EOs which depicted an IC50 of 200 μL L^−1^.^210^ On the other hand, the SLN/LaEO nanosystem exhibited an IC50 of 122 ± 11 μL L^−1^ against HCT-116 cells, compared to free *L. alba* EOs which depicted an IC50 of 145 μL L^−1^. Additionally, SLN/LaEO exhibited favorable cytotoxic effects against A549 cancer cells with an IC50 of 131 ± 8 μL L^−1^, compared to free *L. alba* EOs which showed an IC50 of 275 μL L^−1^.^210^ Moreover, the SLN/CnEO nanosystem showed more than 90% viability in normal WI-38 cells, using concentrations of 50 and 100 μL L^−1^, indicating a favorable safety profile.^210^ Also, these findings were further supported by assessing the hemotoxicity of SLN/CnEO which revealed low hemolytic ratios of less than 3.5% for erythrocytes treated with high concentrations of 100, 200, and 400 μL L^−1^ of SLN/CnEO after 48 h treatment, establishing biomedical use standards. In contrast, free *C. nepeta* EOs exceeded a 5% hemolytic ratio at 400 μL L^−1^, suggesting that the SLN/CnEO nanosystem could enhance the safety profile of the encapsulated EOs while securing lower toxic effects.^210^ Thus, the prepared nanosystems showed enhanced cytotoxic activity against cancer cells while maintaining a favorable safety profile, highlighting their potential as therapeutic agents for cancer treatment.

Dousti *et al.* (2022) could successfully enhance the anticancer potency of *Pistacia atlantica* EOs against breast cancer MDA-MB-231 cells, by incorporating them into SLN.^211^ The chemical composition of the extracted *P. atlantica* EOs was analyzed *via* GC-MS, revealing α-pinene (95.06%) to be the major compound.^211^ The obtained SLN exhibited a diameter of 97.9 ± 1.0 nm, PDI of 0.40 ± 0.03, *Z*-potential of −9.9 ± 1.2 mV, and EE% of 97.3%, suggesting excellent stability and a homogeneous profile of the nanoparticles.^211^ The anticancer activity of SLN encapsulating EOs was assessed against MDA-MB-231 cancer cells, demonstrating a significant reduction in cell viability to 22.13% ± 2.35, using a concentration of 50 μg mL^−1^, after 24 h incubation, and to 26.64% ± 0.93, using a concentration of 1 μg mL^−1^, following 48 h of cell treatment, compared to free EOs which failed to reveal a significant cytotoxic effect. Furthermore, SLN significantly inhibited the proliferation of MDA-MB-231 cells and induced apoptosis, as confirmed by flow cytometric analysis, in which SLN induced significant increases in early apoptotic (Q3) and late apoptotic (Q2) cells with percentages reaching 82.26% ± 1.62 and 10.47% ± 1.25 after 24 h treatment, respectively, compared to 93.06% ± 0.45 and 2.1% ± 0.35 following 48 h treatment, respectively. Additionally, the percentage of necrotic cells (Q1) increased to 2.59% ± 0.2, after 24 h, and to 0.91% ± 0.8, after 48 h.^211^ In addition, the percentages of cells in the subG1 phase, after 24 and 48 h of treatment, were significantly higher in the group treated with SLN encapsulating EOs compared to the placebo group (85.09% ± 1.26 and 54.84% ± 3.3 *vs.* 43.43% ± 1.15 and 58.63% ± 0.99, respectively), representing more cells with fragmented DNA and increased apoptosis of cancer cells.^211^ These findings emphasize the potential of these nanoparticles for future cancer research, paving the way for the discovery of more effective treatments.

Tabatabaeain *et al.* (2022) developed surface-modified SLN encapsulating *Satureja khuzistanica* EOs for targeted cytotoxicity against MCF-7 cancer cells.^212^ The SLN, modified with folate-bound chitosan, exhibited favorable characteristics including a high EE% of 84.5%, size of 279.4 nm, PDI of 0.3, and *Z*-potential of +31.69 mV, indicating a stable and homogeneous nanosystem suitable for therapeutic applications.^212^ Furthermore, the cytotoxicity evaluation of MCF-7 cells treated with the obtained SLN, after 24 h of incubation, revealed an IC50 value of 88.37 μg mL^−1^, compared to a positive control of tamoxifen which showed an IC50 of 34.61 μg mL^−1^. Additionally, the SLN encapsulating *S. khuzistanica* EOs showed remarkable safety profile with no cytotoxic effect revealed against HFF normal cells, utilizing various concentrations up to 125 μg mL^−1^.^212^ Flow cytometric analysis of the MCF-7 cells treated with SLN encapsulating the EOs showed a promising dose-dependent escalation in the percentage of cells in the Sub-G1 phase. The number of cells in the Sub-G1 phase suggests cell death by apoptosis in the cell cycle analysis. Hence, the MCF-7 treated cells with SLN showed a significant increase in the percentages of the apoptotic cells in the Sub-G1 phase reaching 26.7%, 45.8%, and 79.8%, utilizing concentrations of 31.2 μg mL^−1^, 62.5 μg mL^−1^, and 125 μg mL^−1^, respectively, compared to approximately only 8% of cells in Sub-G1 in untreated cells.^212^ In addition, the molecular analysis further supported the pro-apoptotic activities of the obtained nanoparticles against MCF-7 treated cells, while showing 2.5-fold upregulation of Caspase-9 and a subsequent 3-fold increase in Caspase-3 gene expression. Notably, the elevated Caspase-9 levels suggest cell death through the intrinsic apoptosis pathway, eventually triggering an increased expression of the Caspase-3 gene. Additionally, the positive surface charges of the SLN, attributed to the chitosan coating, likely facilitated enhanced interaction with negatively charged cancer cells, potentially improving intracellular uptake and efficacy.^212^ These findings signify a promising step forward in the development of targeted and effective cancer therapeutics.

### 5.5. Nanostructured lipid carriers

**Fig. 39:**
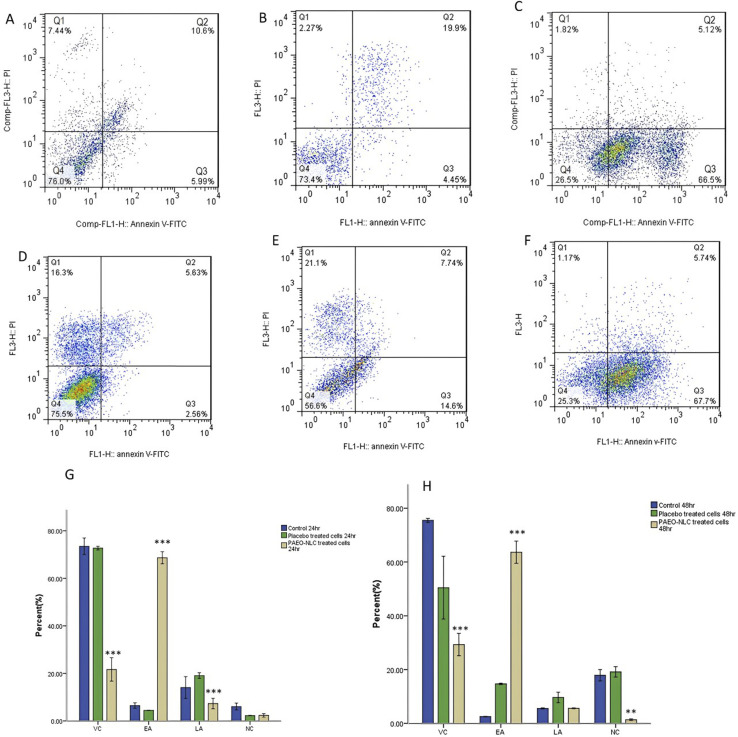
Apoptosis assay of SKBR3 cells treated with nanostructured lipid carriers encapsulating the EOs of *Pistacia atlantica* (PAEO-NLC) (C & F) and placebo (B & E), compared to control cells (A & D), for 24 and 48 h. Panels (G & H) show the quantification of SKBR3 viable (VC), early apoptotic (EA), late apoptotic (LA), and necrotic (NC) cells at 24 and 48 h. Reproduced from ref. 39. Copyright Elsevier 2022.

Najjari *et al.* (2022) synthesized nanostructured lipid carriers encapsulating the EOs of *Pistacia atlantica* to assess their anticancer properties against the breast cancer SKBR3 cells (HER2-positive subtype).^39^ The obtained nanocarriers exhibited a mean size of 151 ± 1 nm, PDI of 0.16 ± 0.03, *Z*-potential of –29.1 ± 1.4 mV, and EE% of 99.3%, reflecting a promising stable profile with remarkable encapsulation efficiency.^39^ More importantly, a significant increase in the cytotoxic activity of the obtained nanocarriers was reported against SKBR3 cells, following 24 and 48 h of cell treatment, with IC50 values of 0.08 μg mL^−1^ and 0.09 μg mL^−1^, respectively. In contrast, free *P. atlantica* EOs failed to show a significant cytotoxic effect on SKBR3 cells, even with a concentration of 50 μg mL^−1^.^39^ Additionally, cell cycle analysis revealed a notable arrest of the treated SKBR3 cells in the Sub-G1 phase, anda mean cell population percentage reached 27.13 ± 1.56%, after 48 h, compared to untreated cells which exhibited a mean population percentage of 5.66 ± 5.40%.^39^ Furthermore, the flow cytometric assay revealed a significant reduction in the percentages of the viable SKBR3 cells (Q4) from 73.46 ± 3.03% and 75.4 ± 0.65% in untreated cells to 21.66 ± 4.27% and 29.3 ± 3.57% in nanocarrier-treated cells, after 24 h and 48 h of incubation, respectively.^39^ Also, the percentages of early apoptotic cells (Q3) increased significantly from 5.99% and 2.56% in untreated cells to 66.5% and 67.7%% in nanocarrier-treated cells, after 24 h and 48 h of treatment, respectively, emphasizing the potemtial of nanocarriers as anticancer agents (Fig. 39).^39^ Further investigation is essential to explore the *in vivo* activity of the obtained nanosystem and to determine the effective drug delivery routes of administration.

### 5.6. Nanofibers

**Fig. 40:**
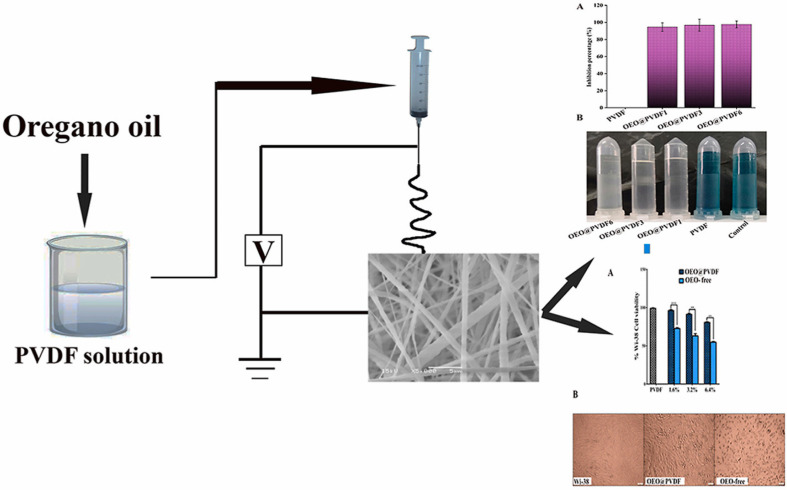
Schematic diagram of oregano EOs electrospun into PVDF nanofibers, showing cytotoxic activity against Huh7 and MDA-MB-231 cancer cells, enhanced stability, higher apoptotic rates, and improved safety against normal cells compared to free EOs. Reprinted with permission from ref. 213. Copyright Elsevier 2022.

El Fawal and Abu-Serie (2022) integrated oregano EOs into polyvinylidene fluoride (PVDF) nanofibers to assess their cytotoxic activity against hepatocellular carcinoma cells (Huh7) and breast cancer cells (MDA-MB-231), as illustrated in Fig. 40.^213^ SEM analysis of the obtained nanofibers revealed an increase in their diameter, from 562 to 770 nm, with increasing concentrations of oregano EO-loaded nanofibers. The cytotoxic evaluation of EO-loaded nanofibers at a concentration of 3.2% (w/v%) against Huh7 and MDA-MB-231 cells showed growth inhibition percentages of 72.4% (*vs.* free EOs 70.70%) and 64.07% (*vs.* free EOs 64.93%), respectively, indicating no significant differences in cytotoxic activity between free EOs and EO-loaded nanofibers.^213^ However, EO-loaded nanofibers at the same concentration exhibited safer toxicity profiles compared to free EOs against Wi-38 normal cells, in which the EO-loaded nanofibers effectively maintained the normal spindle-shaped cell morphology with cell viability exceeding 91.32%, compared to free EOs which exhibited severe morphological changes and a decrease in cell viability to 63.05%.^213^ On the other hand, flow cytometric analysis showed no significant difference between the apoptotic effect of EO-loaded nanofibers (Huh7: 47.19%, MDA-MB-231: 44.74%) and free oregano EOs (Huh7: 44.64%, MDA-MB-231: 42.96%).^213^ Additionally, analysis of apoptotic cells revealed comparable effects, with both free EOs and EO-loaded nanofibers demonstrating equal activation of Nrf2 by ∼4-fold in Huh7 and ∼2-fold in MDA-MB-231 cells, and both upregulating apoptosis-related genes (P53 and Bax) and suppressing NF-κB expression by 6.7 and 5 fold, respectively.^213^ More significantly, the EO-loaded nanofibers exhibited enhanced stability and sustainability, while securing consistent anticancer efficacy compared to free EOs after long-term storage (up to 6 months). After 6 months, EO-loaded nanofibers showed a significant inhibition in Huh7 and MDA-MB-231 cell growth by 71.01% and 63.83%, respectively, compared to 37.68% and 23.19% inhibition shown with Huh7 and MDA-MB-231 cells treated with stored free EOs after 6 months, respectively.^213^ Additionally, flow cytometric analysis revealed induced apoptosis in Huh7 and MDA-MB-231 cells treated with stored EO-loaded nanofibers, with apoptotic population percentages of 43.19 ± 0.375% and 40.89 ± 0.855%, respectively, compared to 14% with stored free EOs, highlighting the crucial role of nanofibers in preserving the activity and stability of EOs.^213^ These findings show the importance of encapsulation of EOs into nanofibers to overcome their instability, volatility, and degradation preserving their anticancer activity over an extended period compared to free EOs.

El Fawal *et al.* (2023) integrated ginger EOs into PVDF nanofibers to evaluate their anticancer potential against hepatocellular carcinoma cells (Huh7) and breast cancer cells (MDA-MB-231).^214^ The resulting nanofibers had diameters ranging from 246–316 nm. The cytotoxic activity of free EOs and EO-loaded nanofibers (at a concentration of 3.2%) against Huh7 and MDA-MB-231 cells showed no significant differences, after 72 h of incubation.^214^ The nanofibers exhibited a growth inhibition of 38.2% against Huh7 cells (compared to 39.6% with free EOs) and 39.3% against MDA-MB-231 cells (compared to ∼39% with free EOs). Additionally, the EO-loaded nanofibers showed safer toxicity profiles compared to free EOs against Wi-38 normal cells, depicting a cell viability of more than 94.48% compared to a decrease to 77.1% induced by free EO treatment. This improvement in cell viability is attributed to the encapsulation of EOs into the nanofibers, which reduced their volatility and toxicity.^214^ Analysis of apoptotic cells revealed comparable effects, with both free EOs and EO-loaded nanofibers demonstrating similar activation of Nrf2 by approximately 4-fold in Huh7 cells and 2-fold in MDA-MB-231 cells, indicating inhibition of cancer progression and maintenance of oxidative balance.^214^ Moreover, both free EOs and nanofiber-loaded EOs upregulated apoptosis-related genes P53 by 3-fold and Bax by 4-fold, while suppressing NF-κB by 0.2-fold, cyclin D by 0.38-fold, and Bcl-2 by 0.4-fold. Nevertheless, the flow cytometric analysis showed no significant difference in the apoptotic effect of EO-loaded nanofibers (Huh7: 80.74%, MDA-MB-231: 63.39%) compared to free ginger EOs (Huh7: 76.30%, MDA-MB-231: 61.93%).^214^ Additionally, the release profile of EOs from the nanofibers showed an increase in ginger EO release from 9.07% at 12 h to 91.60% at 72 h, accompanied by a 6.5-fold increase in antioxidant activity compared to that at the initial concentration.^214^ This enhanced release and antioxidant activity contribute to the potent anticancer effects of EO-loaded nanofibers by scavenging free radicals.

**Fig. 41:**
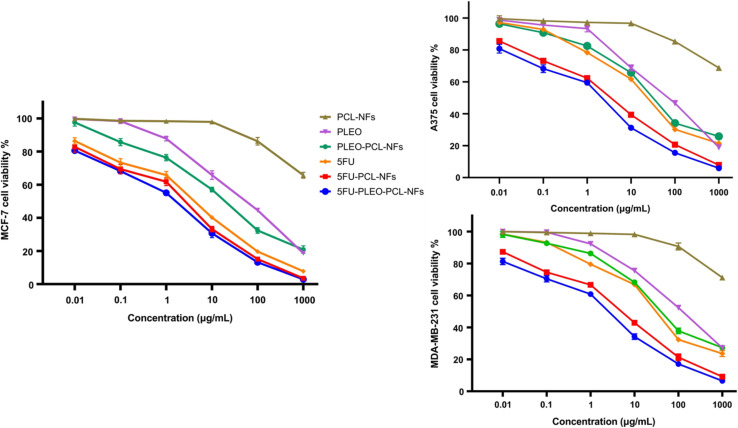
Viability of MCF7, MDA-MB-231, and A375 cancer cells following their treatment with 5-fluorouracil (5FU), free EOs of *Pistacia lentiscus* (PLEO), PCL nanofibers (PCL-NFs), PCL nanofibers loaded with PLEO (PLEO-PCL-NFs), PCL nanofibers loaded with 5FU (5FU-PCL-NFs), and co-loaded PCL nanofibers with 5FU and PLEO (5FU-PLEO-PCL-NFs). Reprinted from ref. 20. Copyright Springer Nature 2024.

EOs extracted from the resins of *Pistacia lentiscus* var. *chia* showed great therapeutic potential. Alabrahim and Azzazy (2024) reported the extraction of *P. lentiscus* EOs *via* hydrodistillation, and determine its chemical composition using GC-MS analysis, showing α-pinene (81.20%) to be the prime component.^20^ Thereafter, *P. lentiscus* EOs and 5FU were loaded into PCL nanofibers (PCL-NFs) *via* electrospinning, to compare and investigate their *in vitro* anticancer activities and their potential synergistic properties against breast cancer (MDA-MB-231 and MCF-7) and melanoma (A375).^20^ The diameter size of the obtained NFs was determined to be between 90.71 nm and 680.95 nm. Nanofiber groups showed greater thermal stability and enhancement of the 5FU thermal stability by at least 1-fold, as determined by thermogravimetric analysis. The nanofibers exhibited exceptional physical integrity throughout 70 days. They maintained robust mechanical and physical properties, demonstrably remaining intact without any dissolution or disintegration observed within their structures.^20^ Furthermore, the biodegradability study showed great stability and prolonged degradation rates of all nanofibers at 42 days with only ∼5% weight loss observed by the end of the study, supporting the effective use of the obtained nanofibers for local therapeutic purposes.^20^ Moreover, the *in vitro* release studies of *P. lentiscus* EOs and 5FU revealed sustainable and prolonged release behaviors over 168 h. Also, higher released amounts of 5FU and *P. lentiscus* EOs were reported (pH 5.4) compared to the physiological media (pH 7.4), indicating higher targeting ability imparted by the loaded nanofibers.^20^ Nanofibers loaded with *P. lentiscus* EOs exhibited significant antioxidant activity with 54.45% free radical scavenging (using DPPH assay).^20^ Finally, the cytotoxicity of free EOs, pristine 5FU, PCL-NFs, 5FU loaded NFs (5FU-PCL-NFs), EOs loaded NFs (EOs-PCL-NFs), and the combination of EOs and 5FU loaded NFs (5FU-EOs-PCL-NFs) was investigated against A375, MDA-MB231, and MCF-7 cancer cells (Fig. 41). For A375 cancer cells, EOs-PCL-NFs showed an IC50 of 15.32 μg mL^−1^ compared to 73.21 μg mL^−1^ for free EOs, and 5FU-PCL-NFs exhibited an IC50 of 7.01 μg mL^−1^ compared to 12.32 μg mL^−1^ for pristine 5FU, whereas 5FU-EOs-PCL-NFs depicted higher cytotoxic activity with an IC50 of 4.24 μg mL^−1^. Furthermore, for MDA-MB-231 cancer cells, EOs-PCL-NFs showed an IC50 of 17.49 μg mL^−1^ compared to 93.58 μg mL^−1^ for free EOs, and 5FU-PCL-NFs showed an IC50 of 9.3 μg mL^−1^ compared to 16.14 μg mL^−1^ for pristine 5FU, whereas 5FU-EOs-PCL-NFs showed higher cytotoxicity with an IC50 of 5.28 μg mL^−1^. Nonetheless, in the case of MCF-7 cancer cells, EOs-PCL-NFs showed an IC50 of 12.10 μg mL^−1^ compared to 67.31 μg mL^−1^ for free EOs, and 5FU-PCL-NFs depicted an IC50 of 5.88 μg mL^−1^ compared to 7.87 μg mL^−1^ for pristine 5FU, whereas 5FU-EOs-PCL-NFs exhibited higher cytotoxic activity with an IC50 of 3.82 μg mL^−1^.^20^ Hence, NFs loaded with *P. lentiscus* EOs and 5FU exhibited greater cytotoxic properties compared to free compounds, and even higher anticancer activity shown with their combined nanofibers. Thus, the developed nanofibers hold potential as a local treatment of breast cancer tissues (post-mastectomy) and melanoma to prevent their possible recurrence.

The following table (Table 3) summarizes various nanosystems utilized for EOs delivery for cancer targeting.

**Table 3:** Nanosystems utilized for EOs delivery for cancer targeting

EOs (plant or composition)	Nanosystem/Preparation method	Cell line\ model	Remarks	Ref.
*Achillea millefolium*	Liposomes and niosomes/thin film hydration	Breast cancer (MCF-7)	Superior cytotoxic activity of niosomes compared to liposomes	184
			Both showed remarkable reduction in cell viability in comparison to free EOs	
Lemongrass	Liposomes/thin film hydration	Breast cancer (SKBR3, MDA-MB-231, and MCF-7)	Doubled cell death and improved apoptosis compared to free EOs	185
*Zingiber officinale*	Liposomes/thin film hydration	Human colon cancer (HT-29)	Lower cytotoxic activity compared to free EOs, explained by the slower and sustained release of the EOs from the liposomes within 24 h only	187
*Origanum vulgare*	Liposomes/thin film hydration	Breast cancer (MCF-7)	Doubled cytotoxic activity compared to free EOs	189
*Syzygium aromaticum* (clove) and *Eucalyptus*	Liposomes/thin film hydration	Mouse skin melanoma (B16-F10)	Reduced cell viability	190
			Co-delivery of EOs and 5FU increased cytotoxicity compared to either of the EOs	
*Rosmarinus officinalis* (Rosemary)	Liposomes/thin film hydration	Breast cancer (MCF-7)	Higher cytotoxic effects compared to free EOs	215
*Origanum vulgare*	Liposomes/ethanol injection technique using different classes of phospholipids (Phospholipon 85G, Lipoid S100, and Phospholipon 90H)	Breast cancer (MCF-7)	Compared to free EOs	216
			Highest cytotoxic activity was shown with cells treated with Phospholipon 90H	
			Insignificant cytotoxic activity was shown with cells treated with liposomes (Lipoid S100)	
			Least cytotoxic impact could be revealed with cells treated with Phospholipon 85G	
*Pistacia lentiscus* variety *chia*	Niosomes/thin film hydration	Breast (MCF-7) and ovarian cancers (Skov-3)	Increased cytotoxicity (by 10-fold) and apoptotic activity compared to free EOs	24
			Augmented upregulation of the proapoptotic markers (Bak and Bax) and downregulation of the antiapoptotic marker (Bcl-2)	
*Pelargonium graveolens* (geranium)	Niosomes/thin film hydration	Breast cancer (MCF-7)	Enhanced cytotoxic and apoptotic effects compared to free EOs	194
			Superior synergistic anticancer activity upon co-encapsulation with ascorbic acid	
*Artemisia vulgaris*	Polymeric PLGA nanoparticles/single emulsion solvent evaporation method	Colon adenocarcinoma cells (HT-29)	Compared to free EOs	195
			Higher cytotoxic and apoptotic activity	
			Superior antiangiogenetic activity (reduced length and number of vessels)	
			Higher selectivity towards malignant cells	
			Safer profile on normal cells	
*Cynometra cauliflora*	Polymeric chitosan nanoparticles/ionic gelation method	Breast cancer (MCF-7 and MDA-MB-231)	Compared to free EOs	196
			More significant selectivity and cytotoxicity against cancer cells	
			No cytotoxicity against normal cells	
*Syzygium aromaticum* (clove)	Polymeric alginate nanoparticles/ionic gelation method	Breast cancer (MCF-7) and melanoma (A-375 cells)	Compared to free EOs	197
			Higher cytotoxic and apoptotic activity (elevation of the Bax/Bcl-2 ratio)	
			Superior anticancer activity in comparison to eugenol loaded nanoparticles	
*Ferula gummosa*	Polymeric alginate nanoparticles/ionic gelation method	Melanoma (A-375) and breast cancers (MDA-MB-231)	Compared to free EOs	198
			Increased cytotoxic activities under hyperoxic conditions (increase in oxygen)	
			Increased oxidative stress	
			Induced apoptosis, Bax upregulation, and Bcl-2 downregulation	
*Boswellia sacra*	Polymeric PLGA–PCL nanoparticles/emulsion solvent evaporation method	Breast cancer (MCF-7)	Higher cytotoxic, apoptotic, and necrotic activities compared to free EOs	199
			Induced cell cycle arrest at the G0–G1 phase	
*Aegle marmelos*	Polymeric chitosan nanoparticles/ion gelation method	Lung cancer cells (A549)	Increased cytotoxicity and apoptotic activity (by two-fold), compared to free EOs	202
			Increased necrotic cells, chromatin condensation, and decreased cell division evidenced by phase contrast microscopy	
*Laurus nobilis*	Polymeric PLGA nanoparticles/single emulsion solvent evaporation method	DNA binding assay and molecular docking (PI3K/mTOR)	Induced hydrogen bonding with the dual inhibitor of PI3K/mTOR and alterations in the electronic structure of DNA (6 nm bathochromic shift and 93.80% hypochromic effect), reflecting DNA damage induction and hence cell death	203
*Boswellia sacra*	Nanoemulsions/low energy emulsification technique	Lung cancer (A549)	Upregulated Bax, P53, Caspase 8, FAAD, and DR52	204
			Downregulated STAT-3, NF-kB, and Bcl-2	
			Increased levels of nitric oxide and inducible nitric oxide synthase enzyme	
			Induced higher cytotoxic activity in comparison to free EOs	
*Ferula assa-foetida*	Nanoemulsions/emulsification technique using a probe sonicator	Melanoma (A-2058), breast cancer (MCF-7), and *in vivo* murine breast cancer model (Balb/C mice)	Upregulated Bax expression	205
			Downregulated Bcl-2, VEGF and VEGFR expression (anti-angiogenesis)	
			Higher apoptotic and cytotoxic activities against MCF-7 compared to A-2058	
			Excellent safety margin profile against normal cells	
			Reduced murine breast tumor size (*in vivo* assessment)	
*Ferula gummosa*	Nanoemulsions/emulsification technique using ultrasonic waves	Colon cancer (HT-29) and *in vivo* murine colon cancer model (male Balb/C mice)	Upregulated CAT, SOD, caspase-3, Bax, and caspase-9 expression	206
			Downregulated Bcl-2 and VEGF expression (anti-angiogenesis)	
			Excellent cytotoxic and apoptotic activities	
			No inhibitory effects against normal cells	
			Reduced tumor volume (two-third) in the murine breast cancer model (*in vivo* assessment)	
*Syzygium aromaticum* (clove)	Nanoemulsions/emulsification technique using ultrasonic waves	Colon cancer (HT-29) and *in vivo* female Balb/C mice	Higher cytotoxic and apoptotic activities compared to free EOs	207
			Increased caspase-3 expression (5-fold)	
			Safe profile evidenced by the absence of *in vivo* histopathological changes in the intestine, liver, and kidney	
			Increased expression of antioxidant genes SOD (3.5×), CAT (2.4×), and GPx (1.8×)	
*Mentha pulegium*	Nanoemulsions/low energy emulsification technique	Melanoma cells (A375)	Superior anticancer activity of the prepared nanogel compared to the nanoemulsion	208
	Nanogel/crosslinking of nanoemulsions		Better early apoptosis induction of the prepared nanoemulsion compared to the nanogel	
			Upregulation of Bax and downregulation of Bcl-2 in both nanosystems	
*Pistacia atlantica*	Nanoemulsions/emulsification technique using ultrasonic waves	Prostate cancer (PC3), lung cancer (A549), melanoma cells (A2058), and *in vivo* Ross fertilized chicken eggs	Superior cytotoxic effect against melanoma cells, compared to prostate and lung cancer cells	209
			Upregulated caspase-3, caspase-8, and IL-10 in all cancer cells	
			Reduced blood vessels (number and length) and chick embryos (height and weight) in *in vivo* assessment	
*Origanum glandulosum* Desf.	Nanocapsules/high-speed homogenization method	Hepatocellular carcinoma (HepG2)	Higher cytotoxic activity of the prepared nanocapsules, compared to prepared nanoemulsions and free EOs	188
	Nanoemulsions/high pressure homogenization technique			
*Mentha piperita*	Nanoemulsion/spontaneous emulsification method	Breast cancer (MCF-7, MDA-MB-231, and MDA-MB-468)	Greater cytotoxicity against all breast cancer cell lines compared to free EOs	217
*Lavandin*	Nanoemulsions/solvent displacement–evaporation technique with spontaneous emulsification	Human neuroblastoma (SH-SY5Y), human breast cancer (MCF-7), human lymphoblastic leukemia (CCRF CEM), and human colorectal adenocarcinoma (Caco-2)	Enhanced cytotoxicity against all cell types, especially Caco-2, compared to free EOs	218
			Increased modifications in mitochondria and apoptotic microtubule networks in Caco-2 cells (TEM images)	
*Zataria*	Nanoemulsions (modified citrus pectin)/solvent dissolution and homogenization method	Breast cancer (MCF-7, MDA-MB-231, and T47D cells) and (MDA-MB-231 spheroids)	G2/S phase cell cycle arrest	219
			Increased interaction with DNA, reflecting higher DNA damage and cell death (groove binding/partial intercalative complex)	
			Higher ROS production and mitochondrial membrane disruption, increasing apoptosis in MDA-MB-231 cells and spheroids	
			Greater cytotoxic activity compared to free EOs	
*Zataria multiflora*	Nanoemulsions (modified apple pectin)/electrospraying method	Breast cancer (MCF-7), (MDA-MB-231), and (T47D)	Compared to free EOs	220
			Superior DNA interaction	
			Greater ROS production, cell cycle arrest at the G2/M phase, cytotoxicity, and mitochondrial apoptotic activity	
*Anethum graveolens*	Nanoemulsions/ultrasonication method	Lung cancer (A549)	Greater cytotoxicity and apoptotic activity against tumor cells	221
			Safer profile against normal cells	
			Upregulated apoptotic gene expression (caspase-3 and caspase-8)	
*Citrus aurantium*	Nanoemulsions/ultrasonication method	Lung cancer (A549)	Higher cytotoxicity and apoptotic activity	222
			Sub-G1 cell cycle arrest	
			Overexpression of caspase-3 (∼4×)	
			No *in vivo* histopathological differences in the intestine, liver, kidney	
			Increased expression of antioxidant genes: SOD (3.9×), CAT (2.1×), and GPx (2.8×)	
			Hepatic antioxidant redox potential was also improved	
*Jasminum humile* and *Jasminum grandiflorum*	Nanoemulsions/spontaneous emulsification method	Breast cancer (MCF-7), hepatocellular carcinoma (HepG2), and leukemia (THP-1)	Potent cytotoxic activity, especially against HepG-2 cells, compared to free EOs and a standard drug (Doxorubicin)	223
*Santolina chamaecyparissus*	Nanoemulsions/aqueous phase titration method	Breast cancer (MCF-7), colorectal adenocarcinoma (Caco-2), and hepatocellular carcinoma (HepG2)	Higher cytotoxicity compared to a standard drug (gemcitabine), except for Caco-2 cells	224
*Pulicaria crispa*	Nanoemulsions/high-pressure homogenization process combined with the phase inversion temperature	Breast cancer (MCF-7) and hepatocellular carcinoma (HepG2)	Synergistic cytotoxic effect (100×) and greater apoptotic activity against MCF-7 (4.48×) and Hep-G2 (2.95×) for the co-loaded (EOs + gemcitabine) nanoemulsions, compared to gemcitabine alone	225
			Upregulation of P53 and caspase-3	
*Artemisia vulgaris*	Nanoemulsions/ultrasonication method	Breast cancer (MCF-7)	Greater cytotoxicity and apoptotic activity, compared to free EOs	226
			Upregulation of caspase-9, CAT, and SOD expression	
			Downregulation of the VEGF (anti-angiogenesis) in the chick embryo chorioallantoic membrane	
*Zingiber ottensii*	Nanoemulsions/high-pressure homogenization method	Cervical cancer (HeLa), leukemia (K562), lung cancer (A549), and breast cancer (MCF-7)	Enhanced cytotoxicity, especially against MCF-7 followed by cervical, lung and leukemia cells, respectively, when compared to free EOs	227
	Nanoemulgels/addition of a gelling agent to a previous nanoemulsion		Nano- and micro- emulsions were superior in cytotoxic activity compared to nano- and micro-emulgels (gelling agents increased droplet sizes and might prevent the penetration of EOs to cancer cells easily)	
	Microemulsion/high-pressure homogenization method with a surfactant mixture (ethyl alcohol and tween 80)			
	Microemulgels/addition of a gelling agent to a previous microemulsion			
*Heracleum persicum*	Nanoemulsions/ultrasonication method	Breast cancer (MDA-MB-231)	Decreased cancer cell viability and migration significantly, arresting at sub-G1, compared to normal cells	228
			Upregulated caspase-3 (7.8×) expression and antioxidant genes SOD (2.8 ×), CAT (2.3×), and GPx (1.9×)	
			Safe profile evidenced by the absence of *in vivo* histopathological changes in the intestine, liver, and kidney	
*Pinus morrisonicola*	Nanoemulsions/ultrasonic emulsification method	Lung cancer (A549) and hepatocellular carcinoma	Greater cytotoxicity and apoptotic activity against colon, followed by prostate, breast, lung, and liver cancer cells	229
		(HepG2), breast cancer (MCF-7), prostate cancer (PC3), and colon cancer (HT-29)	Safe profile against HFF normal cells	
			Cell cycle arrest at the Sub-G1 phase and overexpression of caspase-3, caspase-9, VEGF, VEGFR, CAT, and SOD in HT-29 cells	
*Teucrium polium*	Nanoemulsions/high-pressure homogenization method	Colon cancer (HCT116, wild type p53) and (HT-29, mutant-type P53)	Synergistic apoptotic effect of co-loaded EOs + Oxaliplatin and greater cytotoxic activity against HCT116 cells (1.4×) and HT-29 (1.3×) compared to EO-loaded nanoemulsions alone	230
			Elevated levels of ROS, Bax, and P53	
			Induced necrosis and apoptosis	
*Carum Carvi*	Nanoemulsions/ultrasonication method	Colon cancer (HT-29)	Prominent cytotoxic and apoptotic effects	231
			Cell cycle arrest at Sub-G1	
			Upregulation of caspase 3 (4.5×) expression	
			Safe profile against normal cells	
*Nigella sativa*	Nanoemulsions/spontaneous emulsification method	Hepatocellular carcinoma (HepG2) and (Huh-7)	Greater antiproliferative and apoptotic activities (higher against Huh-7 cells), compared to free EOs and a standard drug (Doxorubicin)	232
			Upregulated Bax expression and downregulated Bcl-2 expression	
			Insignificant cytotoxic effects against normal cells	
*Saccocalyx satureioides Coss. et Durieu*	Nanoemulsions/high-pressure homogenization method	Hepatocellular carcinoma (HepG2)	Stronger cytotoxic effect when compared to free EOs	233
			Lower antioxidant activity compared to free EOs (correlated with variations in total flavonoid, phenolic, and volatile compounds)	
*Ricinus communis*	Nanoemulsions/high- energy homogenization followed by ultrasonication method	Hepatocellular carcinoma (HepG2)	Decreased HepG2 cell viability remarkably	234
			Induced cell cycle arrest at the G0/G1 phase (increased sub-G1 cells)	
			Upregulated caspase-3 (3.23×)	
*Cuminum cyminum*	Nanoemulsions/ultrasonication method	Tongue carcinoma (SAS)	Higher cytotoxicity and selective cancer cell targeting	235
			Increased early apoptotic activity	
			Safe profile observed on normal cell lines	
Celery	Nanoemulsions/ultrasonication method	Tongue carcinoma (SAS)	Superior cytotoxic and antiproliferative effects, compared to free EOs	236
			Increased apoptotic activity (∼27%)	
*Clinopodium nepeta* and *Lippia alba*	Solid lipid nanoparticles/homogenization by the ultrasonication method	Human lung cancer (A549) and colon cancer (HCT-116)	Enhanced cytotoxic activities shown with both nanosystems, compared to their corresponding free EOs	210
			Superior cytotoxic activity against lung cancer cells (SLNs encapsulating *C. nepeta* EOs)	
			Greater cytotoxic activity against colon cancer cells (SLNs encapsulating *L. alba* EOs)	
*Pistacia atlantica*	Solid lipid nanoparticles/probe-ultrasonication	Breast cancer (MDA-MB-231)	Remarkable reduction in cell viability and increase in apoptotic activity, compared to free EOs	211
			Cell cycle arrest at the Sub-G1 phase (fragmented DNA)	
*Satureja khuzistanica*	Solid lipid nanoparticles/high-pressure homogenization method	Breast cancer (MCF-7)	Greater cytotoxic and apoptotic activities	212
			Increased cell cycle arrest in the Sub-G1 phase	
			Increased upregulation of caspase-9 (2.5×) and caspase-3 (3×) gene expression, compared to untreated cells	
			Safe profile against normal cells	
*Mentha longifolia* and *Mentha pulegium*	Solid lipid nanoparticles/high-pressure homogenization method	Breast cancer (MDA-MB-468), (MCF-7) and melanoma (A-375)	*M. pulegium* loaded SLNs and *M. longifolia* loaded SLNs exhibited significant cytotoxic activities against A-375 (∼95%) and MCF-7 (∼93%), respectively, compared to both free EOs	237
			Great cytotoxic activity of both nanosystems shown against MDA-MB-468 (∼85%), compared to their free EOs	
*Foeniculum vulgare*	Solid lipid nanoparticles/high-energy homogenization followed by ultrasonication method	Breast cancer (MCF-7)	Higher cytotoxicity and apoptotic activity, compared to free EOs	238
			Increased Sub-G1 cell cycle arrest (elevated apoptosis)	
*Zataria multiflora*	Solid lipid nanoparticles/high- energy homogenization followed by ultrasonication method	Breast cancer (MDA-MB-468) and melanoma (A-375)	Superior anticancer and antiproliferative effects compared to free EOs	239
*Pistacia atlantica*	Nanostructured lipid carriers/probe-ultrasonication	Breast cancer (MCF-7)	Increased cytotoxicity and early apoptotic cell activity (greater cell cycle arrest in the Sub-G1 phase) in comparison to free EOs	39
Oregano	PVDF nanofibers/electrospinning	Hepatocellular carcinoma (Huh7) and breast cancer (MDA-MB-231)	No difference in cytotoxic and apoptotic activities between free EOs and loaded nanofibers	213
			Excellent safety profile against normal cells	
			Superior apoptotic and cytotoxic activity of stored loaded nanofibers, compared to free stored EOs, indicating the role of nanofibers in preserving the activity and stability of EOs (storage period: 6 months)	
Ginger	PVDF nanofibers/electrospinning	Hepatocellular carcinoma (Huh7) and breast cancer (MDA-MB-231)	No difference in cytotoxic and apoptotic activities between free EOs and the loaded nanofibers	214
			Greater safety profile against normal cells	
			Superior antioxidant activity (6.50×) of the loaded nanofibers, following 72 h of incubation	
*Pistacia lentiscus* var. *chia*	PCL nanofibers/electrospinning	Breast cancer (MDA-MB-231 and MCF-7) and melanoma (A-375)	Higher cytotoxic activities against cancer cells when compared to free EOs, and even superior synergistic anticancer activity when combined with 5FU	20

## 6. Conclusions and future perspectives

Combating cancer remains a pressing global health concern. While chemotherapy continues to be the prime approach of treatment for various cancers, its effectiveness is often surpassed by severe side effects and long-term organ damage. This has driven the search for alternative treatment approaches with improved safety profiles. Essential oils (EOs), entailing a rich library of diverse and potent bioactive compounds, such as terpenoids, monoterpenes, sesquiterpenes, and other aromatic compounds, have emerged as promising candidates in this quest, showing promising anticancer and other therapeutic effects. However, limitations such as poor stability, limited bioavailability, volatility, and non-specific targeting have hampered the therapeutic potential of EOs.

Nanosystems were introduced as efficient delivery platforms for EOs to overcome their limitations paving the way for a more targeted and effective approach to cancer therapy. Hence, the application of nanocarriers encapsulating EOs in cancer treatment holds great potential. Meanwhile, several challenges such as ensuring the *in vivo* stability and sustained release of nanocarriers encapsulating EOs, minimizing side effects, and optimizing large-scale production for clinical use remain to be further addressed. Also, safety and efficacy profiles of the proposed novel nanoformulations need rigorous preclinical and clinical studies.

In the pursuit of personalized medicine, researchers could explore formulating patient-specific combinations of EOs or utilize genetic profiles to guide treatment selection for each patient's specific needs. Additionally, research on stimuli-responsive nanocarriers for targeted drug release at the tumor site holds immense promise. These nanocarriers can be fabricated to respond to specific external stimuli (*e.g.*, changes in pH, temperature, and light, or specific enzymes), allowing for controlled release of EOs when they reach the tumor microenvironment. This targeted approach can significantly minimize systemic side effects and improve therapeutic efficacy.

Furthermore, the field of nanomedicine is constantly evolving, with exciting new avenues opening up for exploration. Biomimetic nanocarriers, for instance, are being designed to mimic the natural behavior of biological systems. These carriers can potentially evade the immune system and deliver their cargo(s) directly into cancer cells with even greater efficiency. Another promising research area lies in the development of combination therapies utilizing EO-loaded nanocarriers alongside other therapeutic modalities, such as chemotherapy or immunotherapy. By combining these approaches, researchers hope to achieve synergistic effects and overcome resistance mechanisms, leading to more effective cancer treatment strategies.

As such, the integration of the advancements in nanotechnology with a deeper understanding of EO bioactivity should allow researchers to reveal the full potential of this exciting field for the development of highly needed, efficacious, and personalized cancer therapies. This could significantly improve patient outcomes and pave the way for a new era of targeted and effective cancer treatment.

This review comprehensively explored the potential of nanocarriers loaded with EOs as a promising approach for cancer therapy. EO extraction methods, chemical composition, mechanisms of action, and targeting capabilities were highlighted supporting their potential as anticancer agents. Furthermore, the diverse landscape of nanocarriers, and their advantages and considerations for EO encapsulation and cancer targeting were highlighted.

EO-loaded nanocarriers offer several significant advantages over traditional free EOs, including enhanced cellular uptake, controlled drug release, and improved therapeutic efficacy. These findings support the potential of EOs loaded into nanosystems for revolutionizing cancer treatment.

While confirming previous findings regarding the anticancer properties of EOs and the benefits of nanocarrier-based delivery systems, this review introduces novel perspectives. It highlights the potential of personalized medicine approaches and the use of stimuli-responsive nanocarriers for targeted drug release. These advancements offer exciting possibilities for improving the efficacy and safety of EO-loaded nanocarriers in cancer therapy.

In conclusion, nanocarriers loaded with EOs represent a novel approach for cancer treatment. While challenges remain, such as ensuring *in vivo* stability and minimizing side effects, the advancements in nanotechnology and our understanding of EO bioactivity provide a strong foundation for future research and clinical translation.

## Data availability

No primary research results, software or code have been included and no new data were generated or analyzed as part of this review.

## Conflicts of interest

The authors declare no competing interests.
